# Supplementary material for: Digitalizing the community health information system improved women’s retention on the maternal continuum of care pathway in northwest Ethiopia: A quasi-experimental study
Source: PLoS One. 2025 Mar 10;20(3):e0312965. doi: 10.1371/journal.pone.0312965 (PMC11892869; doi:10.1371/journal.pone.0312965)
Supplement: Attachment — Submitted filename: point by point response.pdf [file pone.0312965.s002.pdf]

Point-by-point response to previous submission

Editor comments

	Comments	Authors response
1	Aspects of the study are not well described, including the study design and analysis methods	Thank you so much dear editor for the comments. The study design utilized in this study was mentioned on page 5, line 138-141 and the analysis method carried out was also found in page 8, line 238-244.
**2**	Data are presented in tables in ways that are difficult to follow	Thank you so much dear editor for the comments. This study was a pre-post study design and included participants at both points before and after the intervention. Data in the table were to show the participants characteristics particularly on the study variables and the numbers in the table were also explained in a textual form. Have a look on page 9 (line257-264); page 10 (line 270-273); page 11 (line 279-281); page 12 (line 285-286), and page 12 (line 292-306). Thank you so much!
**3**	The conclusions reached in the discussion, which focuses significantly on the role of health extension workers (HEW) do not seem to be supported by your results, which reveal that only a minority of participants had any exposure to HEWs.	Thank you dear editor for the comments. The study found that implementation of digital tool at community level using health extension workers (HEWs) was effective and the conclusion was based on this main findings and other covariates of participants found significant during analysis. In Ethiopia, HEWs are the last mile health workers at community level and the digital tool (eCHIS) was also designed and implemented considering their routine community level work architecture. Have a look on page 16, line 383-390. Thank you so much!
**4**	Lastly, if this paper is revised and submitted elsewhere, I strongly recommend a thorough copy edit and perhaps engaging a copy-editor	Thank you dear editor. Since the previous submission of the manuscript, we have been working on it and believe that the manuscript is improved substantially. Hope, we will learn more during revision of the current manuscript and improve it along the paths of publication. Thank you so much!
