# Supplementary material for: Digitalizing the community health information system improved women’s retention on the maternal continuum of care pathway in northwest Ethiopia: A quasi-experimental study
Source: PLoS One. 2025 Mar 10;20(3):e0312965. doi: 10.1371/journal.pone.0312965 (PMC11892869; doi:10.1371/journal.pone.0312965)
Supplement: Attachment — Submitted filename: PONE-D-24-09283_reviewer.pdf [file pone.0312965.s005.pdf]

PLOS ONE

Impact of digitalizing the community health information system on women's retention in the maternal continuum of care pathway in northwest Ethiopia

--Manuscript Draft--

Manuscript Number:	PONE-D-24-09283
Article Type:	Research Article
Full Title:	Impact of digitalizing the community health information system on women's retention in the maternal continuum of care pathway in northwest Ethiopia
Short Title:	Electronic community health informaion system improves maternal continuum of care
Corresponding Author:	Tesfahun Hailemariam<br>UOG: University of Gondar<br>ETHIOPIA
Keywords:	antenatal care; maternal continuum of care; electronic community health information system; health extension workers; Postnatal care; skilled birth attendance; women education; women empowerment, northwest, Ethiopia
Abstract:	Background<br><br>Women's retention on the paths of maternal continuum of care is still a significant challenge for the healthcare system in most developing countries in general and in Ethiopia in particular. Digitalizing primary healthcare using community health workers is one of the flagship agenda of the government of Ethiopia to improve maternal healthcare utilization. However, the evidence on effectiveness of digitalizing community health system on maternal continuum of care using frontline health system workers is uncertain.<br><br>Objective<br><br>To determine the effect of electronic community health information system intervention on maternal continuum of care in northwest Ethiopia.<br><br>Methods<br><br>A pre-post experimental study design was used at Wogera woreda in Northwest Ethiopia. Data were collected from April to May 2022 at the household level. A total of 830 women-children with age less than one-year pairs were included in the study. A log-binomial logistic regression model was used to estimate the effect of electronic community health information system on the maternal continuum of care and its predictors. Relative risk with a 95% confidence interval was reported, and a p-value<0.05 was considered to declare significantly associated factors with retention in the maternal continuum of care.<br><br>Results<br><br>The study revealed that 10.6% and 32.53% (p<.001) of women were retained fully on maternal continuum of care before and after the intervention, respectively. The coverage of maternal continuum of care was 3.12 times higher in the intervention clusters (ARR: 3.12,95%CI: 2.20–4.41). In addition, primary education (ARR: 1.54, 95%CI:1.14, 2.08), secondary and above education (ARR: 1.61, 95%CI:1.08,2.39); pregnancy intention (ARR: 1.67, 95%CI: 1.11,2.51), and autonomy of women in healthcare seeking and decision-making (ARR: 2.02, 95%CI: 1.03, 3.97) were significantly associated with maternal continuum of care.<br><br>Conclusions<br><br>Electronic community health information system significantly contributed the improvement of maternal continuum of care in northwest Ethiopia. Therefore, maternal health service programs in rural areas should consider the implementation of electronic community health information system. Moreover, provision of women's education,

Powered by Editorial Manager® and ProduXion Manager® from Aries Systems Corporation

	prevention of unintended pregnancy, and enhancement of autonomy of women in healthcare and decision-making should be emphasized to improve maternal continuum of care.
**Order of Authors:**	Tesfahun Hailemariam
	Asmamaw Atnafu
	Lemma Derseh Gezie
	Jens Johan Kaasbøll
	Jörn Klein
	Binyam Tilahun
**Additional Information:**	
**Question**	**Response**
**Financial Disclosure**<br><br>Enter a financial disclosure statement that describes the sources of funding for the work included in this submission. Review the submission guidelines for detailed requirements. View published research articles from *PLOS ONE* for specific examples.<br><br>This statement is required for submission and **will appear in the published article** if the submission is accepted. Please make sure it is accurate.<br><br>**Funded studies**<br>Enter a statement with the following details:<br>• Initials of the authors who received each award<br>• Grant numbers awarded to each author<br>• The full name of each funder<br>• URL of each funder website<br>• Did the sponsors or funders play any role in the study design, data collection and analysis, decision to publish, or preparation of the manuscript?<br><br>Did you receive funding for this work?	The author(s) received no specific funding for this work.
**Competing Interests**<br><br>Use the instructions below to enter a competing interest statement for this submission. On behalf of all authors, disclose any competing interests that could be perceived to bias this	The authors have declared that no competing interests exist.

work—acknowledging all financial support and any other relevant financial or non-financial competing interests.<br><br>This statement is required for submission and **will appear in the published article** if the submission is accepted. Please make sure it is accurate and that any funding sources listed in your Funding Information later in the submission form are also declared in your Financial Disclosure statement.<br><br>View published research articles from *PLOS ONE* for specific examples.<br><br>**NO authors have competing interests**<br><br>Enter: *The authors have declared that no competing interests exist.*<br><br>**Authors with competing interests**<br><br>Enter competing interest details beginning with this statement:<br><br>*I have read the journal's policy and the authors of this manuscript have the following competing interests: [insert competing interests here]*<br><br>* typeset	
**Ethics Statement**<br><br>Enter an ethics statement for this submission. This statement is required if the study involved:<br><br>• Human participants<br>• Human specimens or tissue<br>• Vertebrate animals or cephalopods<br>• Vertebrate embryos or tissues<br>• Field research<br><br>Write "N/A" if the submission does not require an ethics statement.	N/A

General guidance is provided below. Consult the [submission guidelines] for detailed instructions. **Make sure that all information entered here is included in the Methods section of the manuscript.**<br><br>**Format for specific study types**<br><br>**Human Subject Research (involving human participants and/or tissue)**<br>• Give the name of the institutional review board or ethics committee that approved the study<br>• Include the approval number and/or a statement indicating approval of this research<br>• Indicate the form of consent obtained (written/oral) or the reason that consent was not obtained (e.g. the data were analyzed anonymously)<br><br>**Animal Research (involving vertebrate animals, embryos or tissues)**<br>• Provide the name of the Institutional Animal Care and Use Committee (IACUC) or other relevant ethics board that reviewed the study protocol, and indicate whether they approved this research or granted a formal waiver of ethical approval<br>• Include an approval number if one was obtained<br>• If the study involved *non-human primates*, add *additional details* about animal welfare and steps taken to ameliorate suffering<br>• If anesthesia, euthanasia, or any kind of animal sacrifice is part of the study, include briefly which substances and/or methods were applied<br><br>**Field Research**<br><br>Include the following details if this study involves the collection of plant, animal, or other materials from a natural setting:<br>• Field permit number<br>• Name of the institution or relevant body that granted permission	
**Data Availability**<br><br>Authors are required to make all data underlying the findings described fully	Yes - all data are fully available without restriction

Powered by Editorial Manager® and ProduXion Manager® from Aries Systems Corporation

available, without restriction, and from the time of publication. PLOS allows rare exceptions to address legal and ethical concerns. See the PLOS Data Policy and FAQ for detailed information.<br><br>A Data Availability Statement describing where the data can be found is required at submission. Your answers to this question constitute the Data Availability Statement and **will be published in the article**, if accepted.<br><br>**Important:** Stating 'data available on request from the author' is not sufficient. If your data are only available upon request, select 'No' for the first question and explain your exceptional situation in the text box.<br><br>Do the authors confirm that all data underlying the findings described in their manuscript are fully available without restriction?	
**Describe where the data may be found in full sentences. If you are copying our sample text, replace any instances of XXX with the appropriate details.**<br><br>• If the data are **held or will be held in a public repository**, include URLs, accession numbers or DOIs. If this information will only be available after acceptance, indicate this by ticking the box below. For example: *All XXX files are available from the XXX database (accession number(s) XXX, XXX.*).<br>• If the data are all contained **within the manuscript and/or Supporting Information files**, enter the following: *All relevant data are within the manuscript and its Supporting Information files.*<br>• If neither of these applies but you are able to provide **details of access elsewhere**, with or without limitations, please do so. For example:<br><br>*Data cannot be shared publicly because of [XXX]. Data are available from the XXX Institutional Data Access / Ethics*	All relevant data are within the manuscript and its Supporting Information files.

Powered by Editorial Manager® and ProduXion Manager® from Aries Systems Corporation

Committee (contact via XXX) for researchers who meet the criteria for access to confidential data.<br><br>*The data underlying the results presented in the study are available from (include the name of the third party and contact information or URL).*<br>• This text is appropriate if the data are owned by a third party and authors do not have permission to share the data.<br><br>* typeset	
Additional data availability information:	

Powered by Editorial Manager® and ProduXion Manager® from Aries Systems Corporation

Manuscript
Click here to access/download;Manuscript;Manuscirpt_Po.docx

Impact of digitalizing the community health information system on women's retention in the maternal continuum of care pathway in northwest Ethiopia

Tesfahun Hailemariam[1,2]*, Asmamaw Atnafu[3], Lemma Derseh Gezie[4], Jens Johan Kaasbøll[5], Jörn Klein[6], Binyam Tilahun[1]

Authors' affiliation

[1]Department of Health Informatics, Institute of Public Health, College of Medicine and Health Sciences, University osf Gondar, Gondar, Ethiopia.

[2]Department of Health Informatics, College of Health Sciences, Hawassa, Ethiopia.

[3]Department of Health System and Policy, Institute of Public Health, College of Medicine and Health Sciences, University of Gondar, Gondar, Ethiopia.

[4]Department of Epidemiology and Biostatistics, Institute of Public Health, College of Medicine and Health Sciences, University of Gondar, Gondar, Ethiopia.

[5]Department of Informatics, University of Oslo, Oslo, Norway

[6]Department of Nursing and Health Sciences Campus Porsgrunn, University of South-Eastern Norway, Porsgrunn, Norway

Email address:

TH: tesfahunhailemariam@gmail.com *Correspondence

AA: asme2002@gmail.com

LD: lemmagezie@gmail.com

JJ: jensj@ifi.uio.no

JK: Jorn.Klein@usn.no

BT: binigcms@gmail.com

Abstract

Background: Women's retention on the paths of maternal continuum of care is still a significant challenge for the healthcare system in most developing countries in general and in Ethiopia in particular. Digitalizing primary healthcare using community health workers is one of the flagship agenda of the government of Ethiopia to improve maternal healthcare utilization. However, the evidence on effectiveness of digitalizing community health system on maternal continuum of care using frontline health system workers is uncertain.

Objective: To determine the effect of electronic community health information system intervention on maternal continuum of care in northwest Ethiopia.

Methods: A pre-post experimental study design was used at Wogera woreda in Northwest Ethiopia. Data were collected from April to May 2022 at the household level. A total of 830 women-children with age less than one-year pairs were included in the study. A log-binomial logistic regression model was used to estimate the effect of electronic community health information system on the maternal continuum of care and its predictors. Relative risk with a 95% confidence interval was reported, and a p-value<0.05 was considered to declare significantly associated factors with retention in the maternal continuum of care.

Results: The study revealed that 10.6% and 32.53% (p<.001) of women were retained fully on maternal continuum of care before and after the intervention, respectively. The coverage of maternal continuum of care was 3.12 times higher in the intervention clusters (ARR: 3.12,95%CI: 2.20–4.41). In addition, primary education (ARR: 1.54, 95%CI:1.14, 2.08), secondary and above education (ARR: 1.61, 95%CI:1.08,2.39); pregnancy intention (ARR: 1.67, 95%CI: 1.11,2.51), and autonomy of women in healthcare seeking and decision-making (ARR: 2.02, 95%CI: 1.03, 3.97) were significantly associated with maternal continuum of care.

Conclusions: Electronic community health information system significantly contributed the improvement of maternal continuum of care in northwest Ethiopia. Therefore, maternal health service programs in rural areas should consider the implementation of electronic community health information system. Moreover, provision of women's education, prevention of unintended pregnancy, and enhancement of autonomy of women in healthcare and decision-making should be emphasized to improve maternal continuum of care.

Keywords: Antenatal care; maternal continuum of care; electronic community health information system; health extension workers; postnatal care; skilled birth attendance; women education; women empowerment, northwest, Ethiopia

Background

Globally, an estimated 295000 maternal deaths occurred in 2017, yielding an overall MMR of 211 maternal deaths per 100,000 live births globally, and more than nine out of ten of these deaths (94%) occurred in low- and middle-income countries(277,300 per 100,000 live births) [1]. Sub-Saharan Africa and southern Asia account for up to 86% (254000 per 100,000 live birth). Sub-Saharan Africa alone accounts for 66% (196000 per 100,000 live birth) [1]. Ethiopia has made remarkable gains in reducing maternal mortality over the past several decades, but the total number and rates of these deaths remained too high, and improving maternal health is remaining a major challenge in the health system of the country [2]. The current estimate shows that 401 preventable maternal deaths occur for every 100,000 live births in Ethiopia [1] linked with low maternal continuum of care [3].

Many women die because of complications during and following pregnancy, and most of these complications are preventable [1]. Nearly 40% of women develop complications following delivery, and almost 15% of them encounter potentially life-threatening complications [4, 5]. Poor antenatal and postnatal care attendance accounts for a substantial number of preventable maternal deaths [6]. Currently, countries are striving to reduce unwanted maternal death that occur during pregnancy and childbirth [7, 8]. Moreover, it is the prime focus of sustainable development goals (SDGs) to ensure and sustain women's well-being [9].

The government of Ethiopia has been striving to promote women’s good health through designing policies, strategies, and guidelines at national and local level and implementing for betterment of maternal health programs aligned with sustainable development goals (SDGs) [9, 10]. The long-and short-term training of midwifery nurses and the implementation of guidelines for clinical settings are some of the efforts of the government of Ethiopia to reduce the massive number of maternal deaths.

Even though subsequent maternal healthcare use is recommended to improve maternal health and birth outcomes, as well as to save the lives of women and newborns, healthcare utilization remains a significant challenge in resource-constrained settings in general, and in Ethiopia in particular [2, 11]. The global proportions of women attending at least one antenatal care visit in low-income, middle-income, and industrialized regions were 70%, 68%, and 98%, respectively [12]. The reported proportions of births attended by a skilled birth attendant (SBA) in developing countries and Sub-Saharan regions were 59% and 44%, respectively [8] and

nearly half of pregnant women (48%) in low and middle income countries do not receive the recommended antenatal care visits [13].

In Ethiopia, about 1 out of four women (26%) did not receive any ANC visits [14], and 17% had their first prenatal care visit before the fourth month of pregnancy [15]. Similarly, women who received at least four antenatal care visits were about four out of nine (43%) pregnant women [14]. According to an Ethiopian demographic health survey (EDHS) report [14], the proportion of births attended by SBA is about 48% in Ethiopia. Moreover, despite a large proportion of maternal deaths occur during the first 48 hours after birth [14] and receiving PNC from a healthcare providers at the recommended time with its appropriate contents is recommended to prevent complications that could arise after childbirth, nearly 66% of women do not receive a PNC visit in the first two days after delivery [16]. About 71% of rural women do not receive a postnatal check-up within 48 hours after birth, whereas 52% of urban women do not receive early postnatal check-up [14]. Likewise, the proportion of a continuum of care was variable across different regions in Ethiopia. The pooled proportion of maternal continuum of care in Ethiopia was found to be 25.5% [17]. A study showed that both individual and cluster level factors are determining women healthcare utilization [18].

Digital health has become a solution to improving the health system in the recent years [19-21] and has shown tremendous promise in increasing penetration and better functionality in the healthcare industry. Various studies suggested mobile health with low cost improve health service provided by community health workers [22] as it help them to memorize the service components easily and improve their confidence to use [23]. Moreover, digital health supports community health workers to generate quality health data [24], improve healthcare delivery [25], helps them to be effective in their job aids [26, 27] and improve quality data generation, use service provision [28].

The government of Ethiopia has envisioned digitalizing primary healthcare units through electronic community health information system (eCHIS) program, a high priority area of the information revolution. In eCHIS, HEWs deliver e-CHIS-based job-aids activities using tablet devices and enable them to record, use, and report household data and facilitate referral linkage of pregnant women within health centers and health posts. Though multiple studies have presented the effect of mobile health intervention on the improvement of maternal health service utilization [29-31], the role of trained CHWs involvement in maternal healthcare improvement using electronic community health information system unknown. Understanding

the effect of the eCHIS program on maternal health service could help the government to make further scale-up efforts. Moreover, the study's findings inform policymakers to take intervention measures in improving maternal health program. Therefore, this study aimed to measure the effect of e-CHIS intervention on maternal continuum of care in northwest Ethiopia.

Methods

Study setting and period

This study was conducted at Wogera district northwest Ethiopia (Figure 1). The district has a total number 75 health centers and 404 health posts. During the study period, the number of surviving infants were 8675 [32]. The figure depicted was developed by authors using shape-file from central statistical agency of 2013; map on analysis. The study was conducted at baseline from October to November 2020, and at end-line from April to May 2022.

Figure 1: Map of the study area in the Amhara region, northwest Ethiopia 2022; developed by using shape-file central statistical agency 2013: map on analysis

Study design and Study population

A pre-post experimental study design was employed using baseline and end-line surveys, before and after the intervention. Women who gave birth within the last 12 months preceding the survey and were permanent resident of the intervention district were involved in the study.

Intervention description

To tackle the challenges related to quality health data production, use, and service provision, the government of Ethiopia has taken the initiative to digitize the existing paper-based CHIS through the e-CHIS system. The Amhara regional state health bureau, along with the University of Gondar (UoG), carried out the eCHIS implementation in the Wogera district of northwest Ethiopia. The intervention was skill-oriented training for the implementers on mobile-based community health information system application(Figure 2) followed by woreda led household registration, tablet usage guideline provision, technical support and mentoring, and periodical communications as detailed in (Figure 3).

Figure 2: Electronic community health information system health extension workers Application

During intervention, the following strategies were employed (Figure 3). Provision of mentorship and technical assistance: three supporting team members were assigned and

provided technical assistance for implementers biweekly with local mentor and every two weeks by supporting university throughout the intervention period. In addition, one HIT technician (local mentor) was assigned to provide mentorship and solve eCHIS related problems during implementation.

Communication: Under each cluster, there was a focal person and a midwifery nurse for information exchange and programmatic assistance. A group Telegram channel was created and used as a platform to share information daily in relation to eCHIS progress.

Tablet usage policy: the tablet usage policy was developed both in English and local language by the technical support team from UoG to enable all users to use the tablet devices properly for e-CHIS activities and to ensure the functionality of the system during implementation.

Review meeting: during the entire period, monthly review meetings were conducted in connection with eCHIS implementation amongst the head of the woreda health office, the woreda health office planning officer, the head of the district administration, the head of the zonal health bureau, the head of Amhara regional health bureau planning, and the technical supporting team from UoG.

Figure 3: Electronic community health information system implementation strategies in northwest Ethiopia, 2022

During the implementation, HEWS in the district were trained and performed household and pregnant women registration using customized electronic forms. The HEWs used the tablet for community health data management and service provision using HEWs application (Figure 2). The pregnant women in the intervention cluster were linked with the women's development army using the eCHIS system. Health centers and respective health posts under eCHIS implementation clusters were considered to determine the effect of digitalizing community health information system on maternal continuum of care (Figure 4). The usual or routine activities in the district were HEWs activities using CHIS in the community. We compared the influence of digital component, eCHIS, on maternal health service outcome before and after the implementation of eCHIS.

Figure 4: Intervention clusters and HEWs involved in eCHIS implementation in Wogera district northwest Ethiopia, 2022

Sample size and sampling procedure

The sample size was estimated using the proportion of antenatal care visits four and above which was reported at baseline paper, p1 = 39.6% [32]. Considering the effect size which the intervention could bring change on the interest of outcome, 10% change at intervention district were included during the sample size calculation which gives the proportion two (p2=49.6%) [22]. The level of significance at 5% and 80% power, and one to one ratio between baseline and end-line were considered. Baseline (n1) and end-line (n2) of n2:n1= 1:1, the total sample size was estimated at: n1 = n2 = 404. This sample size estimate is closer to that of the baseline study which was 415, and to maintain better power for the study, the same sample size, i.e. 415 was employed for the end-line study giving a total sample size of 830 which considers the two samples.

We used a multi-stage sampling technique to reach the study participants in the study cluster (Figure 5). Simple random sampling technique was applied to select the health centers in the intervention district and systematic random sampling approach was employed to select the study participants. To get the study sampling frame, we used a family folder which was available at primary healthcare units at health post level and used by HEWs during documentation of pregnant women in the catchment health post [33]. Thus the sampling interval was obtained considering the total sample size estimated and the eligible source population at the district. The study participants were selected systematically using the sampling interval. During data collection, for women who were selected and not available at their house, we gave the waiting time 2-5 days to get them back and include in the study.

Figure 5: Schematic presentation of sampling procedure for study participants before and after the intervention in Wogera District, 2022

Outcome

The dependent variable of the study was maternal continuum of care. Maternal continuum of care was categorized as ‘Yes’ when a woman completes all the required maternal healthcare utilization from antenatal care visit to postnatal care visit within 48 hours after birth. Those women who did not fulfil complete maternal continuum of care components were categorized as ‘No’ [34].

Exposure

Electronic community information system and multiple variables such as age of the respondents; marital status of the respondents; education status of women; education status of their partners; pregnancy intention; membership of community-based health insurance; HEWs' home visits during pregnancy; women's autonomy in healthcare and decision-making at the household; family planning, parity, and complications during pregnancy period were included in the study.

Data collection instrument and procedures

Data collection tool was developed from different literature for this study to measure maternal continuum of care and identify factors associated [34]. The tool was validated using psychometric test such as face and content validity and piloted before actual data collection was commenced. The test result from psychometric properties of content and face validity were in the acceptable range, 98 and 0.81, respectively. The data collectors and supervisors were trained on the data collection tools and procedures. The data were collected via face to face using interviewer administrated questionnaire.

Data management and statistical analysis

Data cleaning and management were employed using Stata 14. Frequency, percentages of the study variables were computed against the outcome of the interest, maternal continuum of care. A bivariable log-binomial logistic regression model was employed to identify the candidate variables for multivariable log-binomial logistic regression analysis at p-value<0.25. Multivariable log-binomial logistic regression analysis was performed by estimating the risk ratios with a 95% confidence interval. A p-value of<0.05 was considered to identify a significant predictor of maternal continuum of care. Multicollinearity was checked using a variance inflation factor. The cutoff point of 10 was used to identify the presence of multicollinearity among predictors [35].

Ethical considerations

Verbal consent was obtained, and participants were informed about the objective and importance of the study; procedure and duration; risk and discomfort; benefits of participating in the study confidentiality, and the right to refuse or withdraw during data collection. Study approval and ethical clearance were obtained from the University of Gondar ethical review board (R.NO. V/P/RCS/05/2020). A formal letter of approval was obtained from Amhara

national regional state health bureau and Central Gondar zonal health department. Informed consent was obtained from all subjects and/or their legal guardian(s). For participants aged <18 verbal informed consent was obtained from their parents and assent was obtained from the minor/participant.

Results

Socio-demographic and reproductive characteristics

A total of 830 women participated in the analysis (n1 = n2 = 415) with a response rate of 100%. The mean (standard deviation, SD) age of participants were 29 and 6.7, respectively, and the minimum and maximum ages were 15 and 49 years, respectively. About 261(31.4%), and 107(12.9%) of women attended primary, and secondary and above education, respectively. The participants who had membership in community-based health insurance were 551 (66.4%). About 650 (78.3%) of women had pregnancy intention for their current child, and 16.2% had autonomy in healthcare-seeking and decision-making. 400(48.2%) of the participants were visited by HEWs during pregnancy lifetime (Table 1).

Table 1: Socio-demographic and reproductive characteristics of the study participants in northwest Ethiopia before and after intervention, 2022(n=830)

Variables	Category	Before intervention n (%)	After intervention n (%)	Total n (%)
Age(years)	15-24	121(14.6%)	102(12.3%)	223(26.9)
	25-34	197(23.7%)	195(23.5%)	392(47.2)
	35 and above	97(11.7%)	118(14.2%)	215(25.9)
Marital status	Married	407(49.0%)	392(47.2%)	799(96.2)
	Others	8(1.0%)	23(2.8%)	31(3.8)
Mother's education	Did not attend education	244(29.4%)	218(26.3%)	462(55.7)
	Primary education	109(13.1%)	152(18.3%)	261(31.4)
	Secondary and above	62(7.5%)	45(5.4%)	107(12.9)
Spouse's education	Did not attend education	261(31.4%)	223(26.9%)	484(58.3)
	Primary education	110(13.3%)	166(20.0%)	276(33.3)
	Secondary and above	44(5.3%)	26(3.1%)	70(8.4)

Pregnancy intention	Yes	328(39.5%)	322(38.8%)	650(78.3)
	No	87(10.5%)	93(11.2%)	180(21.7)
Being insured	Yes	198(23.9%)	353(42.5%)	551(66.4)
	No	217(26.1%)	62(7.5%)	279(33.6)
Role of women in decision making	Husband	4(0.5%)	46(5.5%)	50(6)
	Myself	44(5.3%)	90(10.9%)	134(16.2)
	Jointly (husband and wife)	367(44.2%)	278(33.6%)	645(77.8)
Parity	Para one	99(11.9%)	92(11.1%)	191(23)
	Multipara	316(38.1%)	323(38.9%)	639(77)
History of pregnancy related complications	Yes	72(8.7%)	43(5.2%)	115(13.9)
	No	343(41.3%)	372(44.8%)	715(86.1)
Family planning	Yes	336(40.5)	359(43.3)	695(83.7)
	No	79(9.5)	56(6.7)	135(16.3)

Others: divorced, widow, and separated

Maternal continuum of care

A total of 830 women were participated before and after the intervention (n1 = n2 = 415). About 261(31.4%) and 107(12.9%) of women attended primary and secondary education, respectively. The participants who had autonomy in healthcare-seeking and decision-making at household level were 134(16.2%) (Table 2).

Table 2: Socio-demographic and reproductive characteristics of the study participants in northwest Ethiopia before and after intervention, 2022(n=830)

Variables	Category	Before intervention n (%)	After intervention n (%)	Total n (%)
	15-24	121(14.6%)	102(12.3%)	223(26.9)
Age(years)	25-34	197(23.7%)	195(23.5%)	392(47.2)
	35 and above	97(11.7%)	118(14.2%)	215(25.9)
Marital status	Married	407(49.0%)	392(47.2%)	799(96.2)
	Others	8(1.0%)	23(2.8%)	31(3.8)

Mother's education	Did not attend education	244(29.4%)	218(26.3%)	462(55.7)
	Primary education	109(13.1%)	152(18.3%)	261(31.4)
	Secondary and above	62(7.5%)	45(5.4%)	107(12.9)
Spouse's education	Did not attend education	261(31.4%)	223(26.9%)	484(58.3)
	Primary education	110(13.3%)	166(20.0%)	276(33.3)
	Secondary and above	44(5.3%)	26(3.1%)	70(8.4)
Pregnancy intention	Yes	328(39.5%)	322(38.8%)	650(78.3)
	No	87(10.5%)	93(11.2%)	180(21.7)
Being insured	Yes	198(23.9%)	353(42.5%)	551(66.4)
	No	217(26.1%)	62(7.5%)	279(33.6)
Role of women in decision making	Husband	4(0.5%)	46(5.5%)	50(6)
	Myself	44(5.3%)	90(10.9%)	134(16.2)
	Jointly (husband and wife)	367(44.2%)	278(33.6%)	645(77.8)
Parity	Para one	99(11.9%)	92(11.1%)	191(23)
	Multipara	316(38.1%)	323(38.9%)	639(77)
History of pregnancy related complications	Yes	72(8.7%)	43(5.2%)	115(13.9)
	No	343(41.3%)	372(44.8%)	715(86.1)
Family planning	Yes	336(40.5)	359(43.3)	695(83.7)
	No	79(9.5)	56(6.7)	135(16.3)

Others: divorced, widow, and separated

The study showed that in pre-post comparison, in the intervention district, 44(10.6%) and 135(32.5%) (p<.001) of women were retained fully on the maternal continuum of care (Figure 6).

Figure 6: Maternal health service utilization before and after the intervention of eCHIS in Wogera district, northwest Ethiopia, 2022 (n1=n2=415)

The finding showed that maternal continuum of care in the intervention group increased by 21.9 percentage points compared to comparator group (Table 3).

Table 3: The effect of eCHIS intervention on maternal continuum of care in the intervention district, northwest Ethiopia, 2022

Variables	Category	Maternal continuum of care		Effect size (95%CI)
		Yes n(%)	No n(%)	
Exposure to eCHIS intervention	Yes	135(16.3)	280(33.7)	21.9% (18.9-24.6) *
	No	44(5.3)	371(44.7)	1

* Stands for p-value < 0.001.

Multivariable log-binomial logistic regression analysis

The study showed that maternal education, pregnancy intention, and the autonomy of women for healthcare and decision-making were significantly associated with the maternal continuum of care. After controlling for confounders, women in the intervention group had (ARR: 3.12,95%CI: 2.20–4.41) higher chance of completing the maternal continuum of care compared to women in the comparator group. Women with a primary education level were (ARR: 1.54, 95%CI:1.14, 2.08) more likely to complete the maternal continuum of care than women with no formal education. Similarly, women with secondary and above education levels were (ARR: 1.61, 95%CI:1.08, 2.39) more likely to complete the maternal continuum of care as compared to their counterparts.

Women who were intended to be pregnant with the current child were 1.67 (ARR: 1.67, 95%CI: 1.11, 2.51) more likely to complete the maternal continuum of care than those who had no intention of pregnancy. Moreover, women with decision-making power at household level had 2.02(ARR: 2.02, 95%CI: 1.03, 3.97) higher chance of completing the maternal continuum of care as compared to women who had no decision-making power for healthcare seeking at household level (Table 4).

Table 4: A multivariable log-binomial logistic regression model for predictors of women's completion of maternal continuum of care in northwest Ethiopia, 2022(N=830)

Variables	Category	Maternal continuum of care		Unadjusted RR (95%CI)	Adjusted RR (95%CI)
		Yes n(%)	No n(%)		
Exposure to eCHIS intervention	Yes	135(16.3)	280(33.7)	3.07(2.25,4.19)	3.12(2.20,4.41)
	No	44(5.3)	371(44.7)	1	1
Age group of respondents (years)	15-24	55(6.6)	168(20.2)	1	1
	25-34	86(10.4)	306(36.9)	0.89 (0.66,1.19)	0.99(0.72,1.36)
	35 and above	38(4.6)	177(21.3)	0.72(0.96,1.04)	1.06(0.69,1.62)
Mother's education	Did not attend education	71(8.6)	391(47.1)	1	1
	Primary education	75(9)	186(22.4)	1.87(1.40,2.49)	1.54(1.14,2.08)
	Secondary and above	33(4)	74(8.9)	2.01(1.41(2.86)	1.61(1.08,2.39)
Spouse's education	Did not attend education	85(10.2)	399(48.1)	1	1
	Primary education	74(8.9)	202(24.3)	1.53(1.16,2.01)	1.13(0.85,1.49)
	Secondary and above	50(6)	20(2.4)	1.63(1.07,2.47)	1.26(0.84,1.88)
Pregnancy intended	Yes	156(18.8)	494(59.5)	1.87(1.25,2.82)	1.67(1.11,2.51)
	No	23(2.8)	157(18.9)	1	1
Being insured	Yes	138(16.6)	413(49.8)	1.70(1.24,2.34)	0.90(0.65,1.26)
	No	41(4.9)	238(28.7)	1	1
History of pregnancy related complications	Yes	15(1.8)	100(12)	0.57(0.35,0.93)	0.71(0.44,1.14)
	No	164(19.8)	551(66.4)	1	1
Parity	Para one	54(6.5)	137(16.5)	1.45(1.09,1.90)	1.16(0.83,1.61)
	Multi para	125(15.1)	514(61.9)	1	1
	Husband	8(1)	42(5.1)	1	1

Role of women in decision making	Myself	42(5.1	92(11.1)	1.96(0.99,3.88)	2.02(1.03,3.97)
	Jointly (husband and wife)	129(15.6)	516(62.2)	1.25(0.65,2.40)	1.68(0.87,3.21)
HEWs Home visiting	Yes	99(11.9)	301(36.3)	1.33(1.20,1.73)	0.91(0.68,1.83)
	No	80(9.6)	350(42.2)	1	1
Family planning	Yes	158(19)	537(64.7)	1.46(0.96,2.22)	1.45(0.98,2.15)
	No	21(2.6)	114(13.7)	1	1

Discussion

One in every five women completes the maternal continuum of care at baseline, while one in every three women completes the maternal continuum of care at the study endpoint. Exposure to intervention, women's education, pregnancy intention, and women's autonomy in healthcare and decision-making were associated with the completion of the maternal continuum of care. Our study indicated that women in the intervention group had a higher chance of completing the maternal continuum of care compared to women in the comparator group. The findings agreed with past studies showing that using mobile health involving community health workers have impacted maternal healthcare utilization [25, 28]. The first possible reason for the improvement of the maternal continuum of care in our intervention district could be the fact that HEWs, focal persons, and a midwifery nurse provided maternal health services in integration in which pregnant women had a chance to get registered in the application and receive maternal health service. A digitized HEP program could enable HEWs workers to provide maternal health services and link pregnant women with health facilities. According to the literature, mobile health interventions bring a positive effect on antenatal care, skilled birth attendance, and postnatal care improvement [36], and empowering HEWs [37] yields improved maternal health service utilization [28]. The other possible reason could be the fact that eCHIS interventions might have increased the chance of women to access health education and maternity services that could enable them to adhere to maternal health service utilization. Studies show that mobile health increases adherence to health service utilization, develops health seeking behavior, decreases the chance of missing health care appointments, and enhances client and care provider relationships [38, 39]. The other reason could the connection of women in women development army (WDA), one of the eCHIS functions that could enable women to be agents of health and well-being in the community. The WDA is a structural

arrangement that involves women's development team comprising 1-to-5 connections in the community. In WDA, community members get linked with the primary health care system and easily access actionable health messages [40] that have ability to increase the efficiency of HEWs to refer more women with primary healthcare for maternity care [41].

The finding showed that women education was significantly associated with maternal continuum of care. The current finding was complemented by the studies conducted in Ethiopia [34, 42-44] and elsewhere [45-49]. The association between the completion of maternal continuum of care with education could be explained that educated women could easily understand and take action that delivered from different sources such as health messages from healthcare providers and different medias. In addition, the more a woman educated, the more she could be self-determinant about herself; particularly in seeking healthcare and medical examination and could be able the one to choose by her own without waiting others to decide on her behalf. In addition, cultural influences that could hinder women from getting healthcare service might not affect an educated woman which is a common influencing factor of women not to seek healthcare services in developing countries [50]. A study showed that obeying family members or who have role in family in making decision is considered as a norm or culture which has potential in harming women health seeking behavior [51]. Female education is a proxy indictor to improve maternal healthcare utilization [52] and the more a woman became educated the more she is able to seek healthcare from healthcare provider [53].

Women who had an intention to their pregnancy were more likely to be retained on maternal continuum of care paths as compared to women who had no intention of pregnancy for their current child. This finding is similar with the study findings from Ethiopia [54], and Bangladesh [55]. The possible reason for retention of women on maternal continuum of care among women who had intended pregnancy could be emotional and psychological readiness among who were intended to be pregnant than others. Those who were not ready to be pregnant may not give emphasis to seek care from healthcare providers and even they may not give care for their pregnancy [56]. A study indicated that women who have intention to their pregnancy were more likely to receive maternal healthcare than who have no intention to their pregnancy [57].

Moreover, our study showed that women with the ability to decide or who had a role in making a decision at the household level for healthcare were nearly twofold greater in terms of completing the maternal continuum of care than women whose husbands had a role in making decisions for healthcare-seeking. Our finding is consistent with studies conducted in Ethiopia [44, 58] and abroad [59]. This can be justified by the notion that the more the woman is

autonomous in decision-making, the more she will be able to increase bargaining power with her husband to get maternity care [58]. A study, in this regard, showed that women's autonomy in making personal decisions increases the level of healthcare utilization [60]. Another possible explanation is that the Ethiopian government has been envisioning promoting gender equality through the implementation of different policies and strategies such as providing training on women's empowerment for healthcare decision-making to achieve the 2030 SDG agendas [9, 10].

The study could have limitations related to unobserved sources. Those women who had previous complications related to their pregnancy were not excluded; this could bring social desirability bias. Recall bias may exist in the current study as the participants were asked about the previous healthcare utilization exposure. In this study we applied probability sampling technique to reduce selection bias that could arise during study cluster and participants selection. To minimize the confounding effect, potential variables were included and different statistical approaches were applied during analysis through restricting the eligibility criteria.

Conclusion

One in every three women completed the maternal continuum of care at the study endpoint, which was one in every five women at baseline. The findings suggest that eCHIS intervention is effective in improving the maternal continuum of care. Women's education, pregnancy intention, and women autonomy in healthcare and decision-making for healthcare were factors that influenced women's completion of maternal continuum of care. Therefore, maternal health service programs in rural areas should consider implementation of eCHIS, provision of women's education, prevention of unintended pregnancy, and enhancement of the autonomy of women in healthcare and decision-making to improve the maternal continuum of care.

Acknowledgements

We thank the Amhara national regional state health bureau, Central Gondar zone health department, Wogera district health office, University of Gondar Comprehensive Specialized Hospital, and HEWs for their provision of necessary information and support during data collection. Our gratitude also goes to the study participants, data collectors, and supervisors who took part in the study. This work would not be possible without the financial support from the Doris Duke Charitable Foundation through the University of Gondar.

Declaration

Ethics approval and consent to participate.

The ethical review board of the University of Gondar and the ethical review committee of the Amhara regional health bureau's research and technology transfer office gave the permission and ethical clearance to the study. Permission to conduct the research was sought at all levels of government administration. Each study participant gave their informed consent. To prevent revealing personal identification, data was collected using the codes provided. To avoid unwanted access, the data were stored at the University of Gondar's repository. All methods were carried out in accordance with relevant guidelines and regulations.

Consent for Publication

Not applicable

Availability of data and materials

All relevant data are within the manuscript. The data upon which these findings were developed can also be available upon request.

Competing interests

The authors declare that they have no competing interests.

Funding

This work would not be possible without the financial support from the Doris Duke Charitable Foundation (grant 2017187) through the University of Gondar.

Authors Contributions

Conceptualization: TH, AA, LDG, JJ, JK, BT

Data curation: TH, AA, LDG, JJ, JK, BT

Formal analysis: TH, AA, LDG, JJ, JK, BT

Investigation: TH, AA, LDG, JJ, JK, BT

Methodology: TH, AA, LDG, JJ, JK, BT

Project administration: TH, AA, LDG, JJ, JK, BT

Resources: TH, AA, LDG, JJ, JK, BT

Software: TH

Supervision: TH, AA, LDG, JJ, JK, BT

Validation: TH,

Writing – original draft: TH

Writing – review & editing: TH, AA, LDG, JJ, JK, BT

References

1. WHO U, UNFPA, World Bank Group and the United Nations Population Division. Geneva: World Health Organization; . Trends in maternal mortality 2000 to 2017: estimates 2019. Licence: CC BY-NC-SA 3.0 IGO.
2. Shiferaw S, Spigt M, Tekie M, Abdullah M, Fantahun M, Dinant G-J. The effects of a locally developed mHealth intervention on delivery and postnatal care utilization; a prospective controlled evaluation among health centres in Ethiopia. PloS one. 2016;11(7):e0158600.
3. Yehualashet DE, Seboka BT, Tesfa GA, Mamo TT, Seid E. Determinants of optimal antenatal care visit among pregnant women in Ethiopia: a multilevel analysis of Ethiopian mini demographic health survey 2019 data. Reproductive Health. 2022;19(1):61.
4. Langlois ÉV, Miszkurka M, Zunzunegui MV, Ghaffar A, Ziegler D, Karp I. Inequities in postnatal care in low-and middle-income countries: a systematic review and meta-analysis. Bulletin of the World Health Organization. 2015;93:259-70G.
5. Comfort AB, Peterson LA, Hatt LE. Effect of health insurance on the use and provision of maternal health services and maternal and neonatal health outcomes: a systematic review. Journal of health, population, and nutrition. 2013;31(4 Suppl 2):S81.
6. Nurmatov UB, Lee SH, Nwaru BI, Mukherjee M, Grant L, Pagliari C. The effectiveness of mHealth interventions for maternal, newborn and child health in low–and middle–income countries: Protocol for a systematic review and meta–analysis. Journal of global health. 2014;4(1).
7. Blencowe H, Cousens S, Jassir FB, Say L, Chou D, Mathers C, et al. National, regional, and worldwide estimates of stillbirth rates in 2015, with trends from 2000: a systematic analysis. The Lancet Global Health. 2016;4(2):e98-e108.
8. Organization WH. Strategies towards ending preventable maternal mortality (EPMM). 2015.
9. Assembly UG. 70/1 Transforming our world: the 2030 Agenda for Sustainable Development. A/RES/70/1 New York, United Nations. 2015.
10. Finance Mo, Development E. Ethiopia: 2010 MDGs Report trends and prospects for meeting MDGs by 2015. Ministry of Finance and Economic Development Addis Ababa, Ethiopia; 2010.
11. Feroz A, Perveen S, Aftab W. Role of mHealth applications for improving antenatal and postnatal care in low and middle income countries: a systematic review. BMC health services research. 2017;17(1):1-11.
12. AbouZahr C, Wardlaw T. Antenatal care in developing countries: promises, achievements and missed opportunities-an analysis of trends, levels and differentials, 1990-2001: World Health Organization; 2003.

13. Feroz A, Perveen S, Aftab W. Role of mHealth applications for improving antenatal and postnatal care in low and middle income countries: a systematic review. BMC health services research. 2017;17(1):704.
14. Mini E, Demographic E. health survey 2019: key indicators report. The DHS Program ICF. 2019.
15. Ejigu T, Woldie M, Kifle Y. Quality of antenatal care services at public health facilities of Bahir-Dar special zone, Northwest Ethiopia. BMC health services research. 2013;13(1):443.
16. CSACE I. Ethiopia demographic and health survey 2016. Addis Ababa, Ethiopia, and Rockville, Maryland, USA: CSA and ICF. 2016.
17. Haile D, Kondale M, Andarge E, Tunje A, Fikadu T, Boti N. Level of completion along continuum of care for maternal and newborn health services and factors associated with it among women in Arba Minch Zuria woreda, Gamo zone, Southern Ethiopia: A community based cross-sectional study. PLOS ONE. 2020;15(6):e0221670.
18. Colaci D, Chaudhri S, Vasan A. mHealth interventions in low-income countries to address maternal health: a systematic review. Annals of global health. 2016;82(5):922-35.
19. Krishna S, Boren SA, Balas EA. Healthcare via cell phones: a systematic review. Telemedicine and e-Health. 2009;15(3):231-40.
20. Free C, Phillips G, Galli L, Watson L, Felix L, Edwards P, et al. The effectiveness of mobile-health technology-based health behaviour change or disease management interventions for health care consumers: a systematic review. PLoS med. 2013;10(1):e1001362.
21. Lim MS, Hocking JS, Hellard ME, Aitken CK. SMS STI: a review of the uses of mobile phone text messaging in sexual health. International journal of STD & AIDS. 2008;19(5):287-90.
22. McNabb M, Chukwu E, Ojo O, Shekhar N, Gill CJ, Salami H, et al. Assessment of the quality of antenatal care services provided by health workers using a mobile phone decision support application in northern Nigeria: a pre/post-intervention study. PLOS one. 2015;10(5):e0123940.
23. Thomsen CF, Barrie AMF, Boas IM, Lund S, Sørensen BL, Oljira FG, et al. Health workers' experiences with the Safe Delivery App in West Wollega Zone, Ethiopia: a qualitative study. Reproductive health. 2019;16(1):50.
24. Florez-Arango JF, Iyengar MS, Dunn K, Zhang J. Performance factors of mobile rich media job aids for community health workers. Journal of the American Medical Informatics Association. 2011;18(2):131-7.
25. Mushamiri I, Luo C, Iiams-Hauser C, Amor YB. Evaluation of the impact of a mobile health system on adherence to antenatal and postnatal care and prevention of mother-to-child transmission of HIV programs in Kenya. BMC public health. 2015;15(1):102.
26. Intelligence G. The Mobile Economy: Sub-Saharan Africa 2014. GSMA Intelligence. 2014.

27. Medhanyie AA, Little A, Yebyo H, Spigt M, Tadesse K, Blanco R, et al. Health workers' experiences, barriers, preferences and motivating factors in using mHealth forms in Ethiopia. Human resources for health. 2015;13(1):2.
28. Medhanyie AA, Moser A, Spigt M, Yebyo H, Little A, Dinant G, et al. Mobile health data collection at primary health care in Ethiopia: a feasible challenge. Journal of Clinical Epidemiology. 2015;68(1):80-6.
29. Wagnew F, Dessie G, Alebel A, Mulugeta H, Belay YA, Abajobir AA. Does short message service improve focused antenatal care visit and skilled birth attendance? A systematic review and meta-analysis of randomized clinical trials. Reproductive health. 2018;15(1):191.
30. Gelano TF, Assefa N, Bacha YD, Mahamed AA, Roba KT, Hambisa MT. Effect of Mobile-health on maternal health care service utilization in Eastern Ethiopia: study protocol for a randomized controlled trial. Trials. 2018;19(1):102.
31. Atnafu A, Otto K, Herbst CH. The role of mHealth intervention on maternal and child health service delivery: findings from a randomized controlled field trial in rural Ethiopia. Mhealth. 2017;3.
32. Hailemariam T, Atnafu A, Gezie LD, Tilahun B. Why maternal continuum of care remains low in Northwest Ethiopia? A multilevel logistic regression analysis. Plos one. 2022;17(9):e0274729.
33. Medhanyie A, Spigt M, Kifle Y, Schaay N, Sanders D, Blanco R, et al. The role of health extension workers in improving utilization of maternal health services in rural areas in Ethiopia: a cross sectional study. BMC health services research. 2012;12(1):352.
34. Emiru AA, Alene GD, Debelew GT. Women's retention on the continuum of maternal care pathway in west Gojjam zone, Ethiopia: multilevel analysis. BMC pregnancy and childbirth. 2020;20(1):1-14.
35. Craney TA, Surles JG. Model-dependent variance inflation factor cutoff values. Quality Engineering. 2002;14(3):391-403.
36. Atnafu A, Bisrat A, Kifle M, Taye B, Debebe T. Mobile health (mHealth) intervention in maternal and child health care: Evidence from resource-constrained settings: A review. The Ethiopian Journal of Health Development (EJHD). 2015;29(3).
37. Dusabe-Richards JN, Tesfaye HT, Mekonnen J, Kea A, Theobald S, Datiko DG. Women health extension workers: Capacities, opportunities and challenges to use eHealth to strengthen equitable health systems in Southern Ethiopia. Canadian Journal of Public Health. 2016;107(4-5):e355-e61.
38. Schwebel FJ, Larimer ME. Using text message reminders in health care services: A narrative literature review. Internet interventions. 2018;13:82-104.
39. Rohman L, Maruswezki D, Boyce Cam N. The impact of a text messaging service on orthopaedic clinic Did Not Attend rates. Journal of telemedicine and telecare. 2015;21(7):408-13.

40. Yitbarek K, Abraham G, Morankar S. Contribution of women's development army to maternal and child health in Ethiopia: a systematic review of evidence. BMJ open. 2019;9(5):e025937.
41. Jackson R, Hailemariam A. The role of health extension workers in linking pregnant women with health facilities for delivery in rural and pastoralist areas of Ethiopia. Ethiopian journal of health sciences. 2016;26(5):471-8.
42. Atnafu A, Kebede A, Misganaw B, Teshome DF, Biks GA, Demissie GD, et al. Determinants of the Continuum of Maternal Healthcare Services in Northwest Ethiopia: Findings from the Primary Health Care Project. Journal of Pregnancy. 2020;2020.
43. Fekadu GA, Ambaw F, Kidanie SA. Facility delivery and postnatal care services use among mothers who attended four or more antenatal care visits in Ethiopia: further analysis of the 2016 demographic and health survey. BMC pregnancy and childbirth. 2019;19(1):1-9.
44. Asratie MH, Muche AA, Geremew AB. Completion of maternity continuum of care among women in the post-partum period: Magnitude and associated factors in the northwest, Ethiopia. PLoS One. 2020;15(8):e0237980.
45. Singh K, Story WT, Moran AC. Assessing the continuum of care pathway for maternal health in South Asia and sub-Saharan Africa. Maternal and child health journal. 2016;20(2):281-9.
46. Wang W, Hong R. Levels and determinants of continuum of care for maternal and newborn health in Cambodia-evidence from a population-based survey. BMC pregnancy and childbirth. 2015;15(1):1-9.
47. Shibanuma A, Yeji F, Okawa S, Mahama E, Kikuchi K, Narh C, et al. The coverage of continuum of care in maternal, newborn and child health: a cross-sectional study of woman-child pairs in Ghana. BMJ global health. 2018;3(4):e000786.
48. Hamed A, Mohamed E, Sabry M. Egyptian status of continuum of care for maternal, newborn, and child health: Sohag Governorate as an example. International Journal of Medical Science and Public Health. 2018;7(6):1.
49. Chalise B, Chalise M, Bista B, Pandey AR, Thapa S. Correlates of continuum of maternal health services among Nepalese women: Evidence from Nepal Multiple Indicator Cluster Survey. PLoS One. 2019;14(4):e0215613.
50. Nigatu D, Gebremariam A, Abera M, Setegn T, Deribe K. Factors associated with women's autonomy regarding maternal and child health care utilization in Bale Zone: a community based cross-sectional study. BMC women's health. 2014;14(1):1-9.
51. Bulambo HK. Utilization of Maternal Health Care Services in Rural Mali: The Role of Traditional Values: Emory University; 2012.
52. Teferra AS, Alemu FM, Woldeyohannes SM. Institutional delivery service utilization and associated factors among mothers who gave birth in the last 12 months in Sekela District, North West of Ethiopia: A community-based cross sectional study. BMC pregnancy and childbirth. 2012;12(1):74.

53. Abera M, Belachew T. Predictors of safe delivery service utilization in Arsi Zone, South-East Ethiopia. Ethiopian journal of health sciences. 2011;21(3).
54. Shitie A, Assefa N, Dhressa M, Dilnessa T. Completion and factors associated with maternity continuum of care among mothers who gave birth in the last one year in Enemay district, Northwest Ethiopia. Journal of Pregnancy. 2020;2020.
55. Khan MN, Harris ML, Loxton D. Does unintended pregnancy have an impact on skilled delivery care use in Bangladesh? A nationally representative cross-sectional study using Demography and Health Survey data. Journal of Biosocial Science. 2021;53(5):773-89.
56. Tesfaye G, Chojenta C, Smith R, Loxton D. Predisposing, enabling and need factors associated with skilled delivery care utilization among reproductive-aged women in Kersa district, eastern Ethiopia. Reproductive health. 2019;16(1):1-11.
57. Dutamo Z, Assefa N, Egata G. Maternal health care use among married women in Hossaina, Ethiopia. BMC Health Services Research. 2015;15(1):1-9.
58. Tsegaye B, Shudura E, Yoseph A, Tamiso A. Predictors of skilled maternal health services utilizations: A case of rural women in Ethiopia. Plos one. 2021;16(2):e0246237.
59. Sakuma S, Yasuoka J, Phongluxa K, Jimba M. Determinants of continuum of care for maternal, newborn, and child health services in rural Khammouane, Lao PDR. PloS one. 2019;14(4):e0215635.
60. Alemayehu M, Meskele M. Health care decision making autonomy of women from rural districts of Southern Ethiopia: a community based cross-sectional study. International journal of women's health. 2017;9:213.

Figure

Click here to access/download;Figure;Figure 1.tif

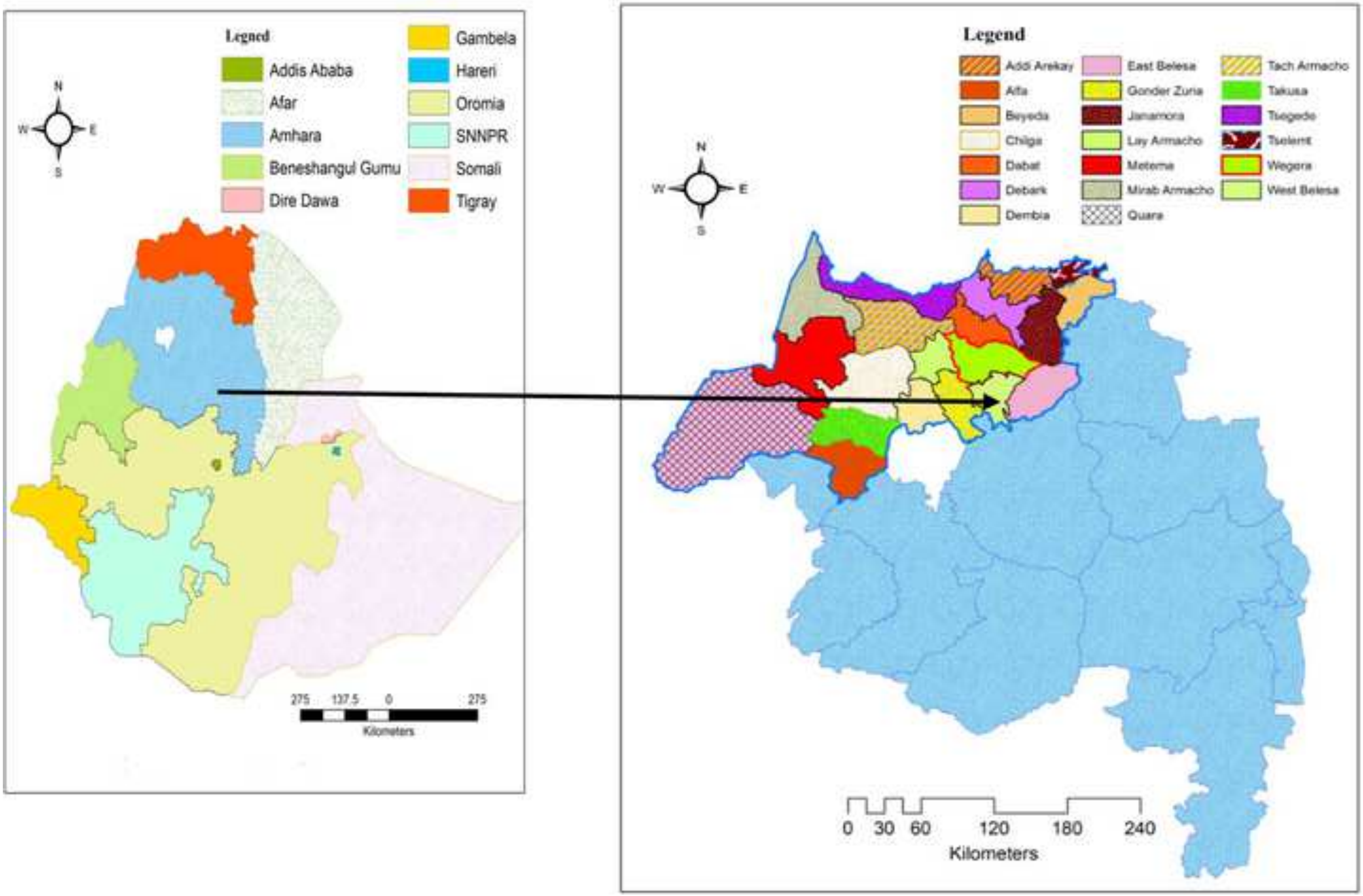

Figure

Click here to access/download;Figure;Figure 2.tif

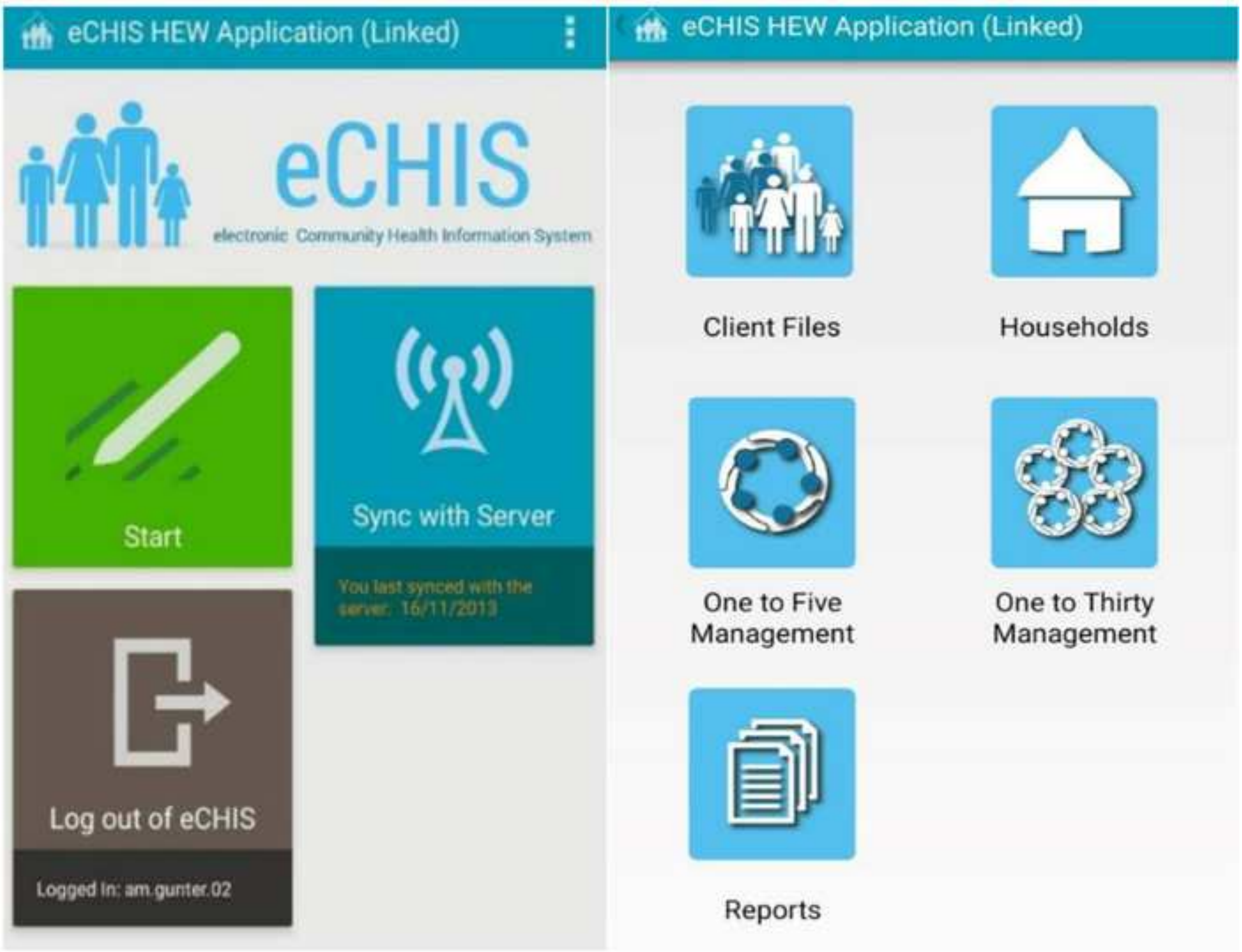

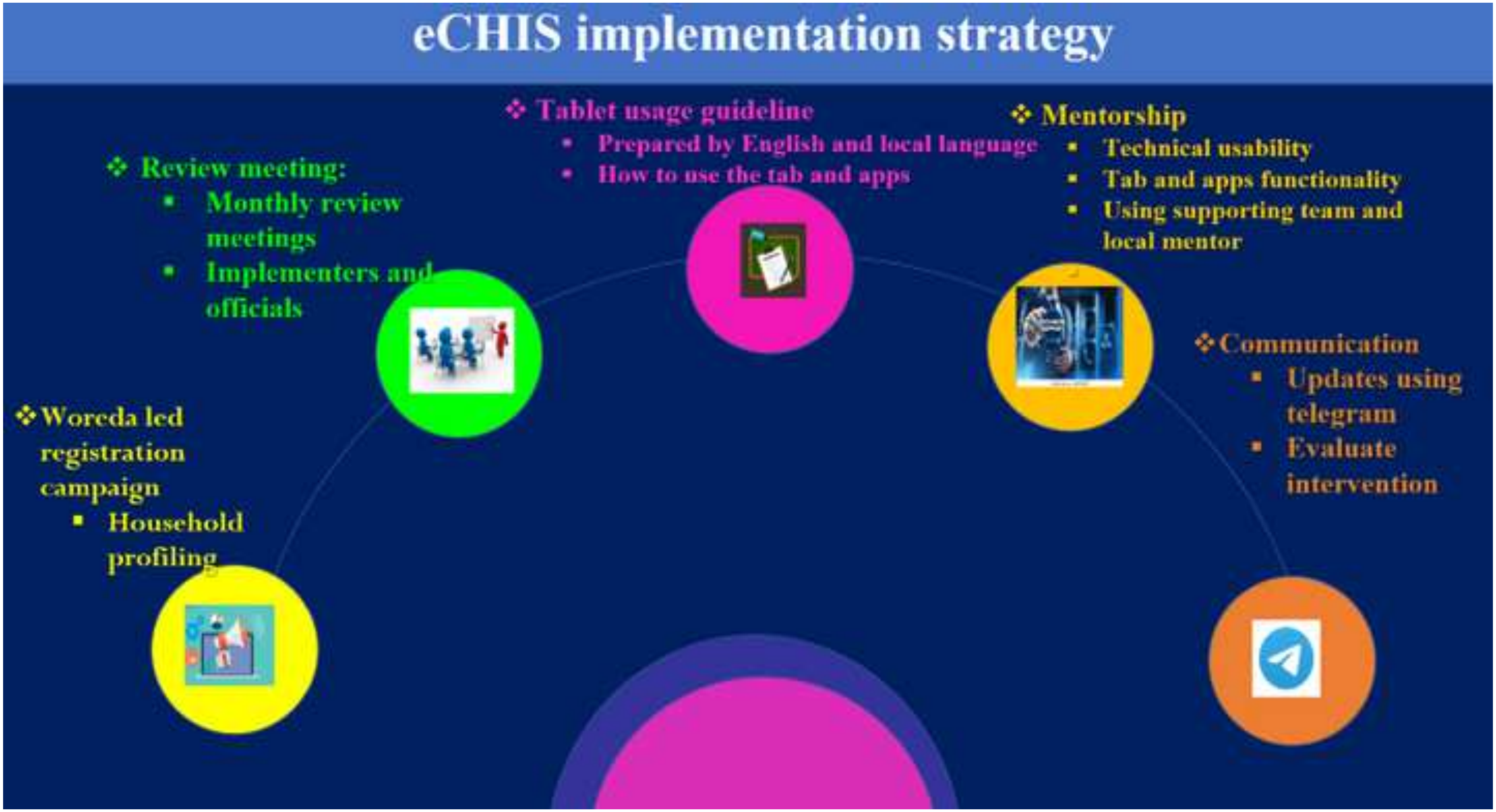
eCHIS implementation strategy
Tablet usage guideline
Prepared by English and local language
How to use the tab and apps
Mentorship
Technical usability
Tab and apps functionality
Using supporting team and local mentor
Review meeting:
Monthly review meetings
Implementers and officials
Communication
Updates using telegram
Evaluate intervention
Woreda led registration campaign
Household profiling

Figure

Click here to access/download;Figure;Figure 4.tif

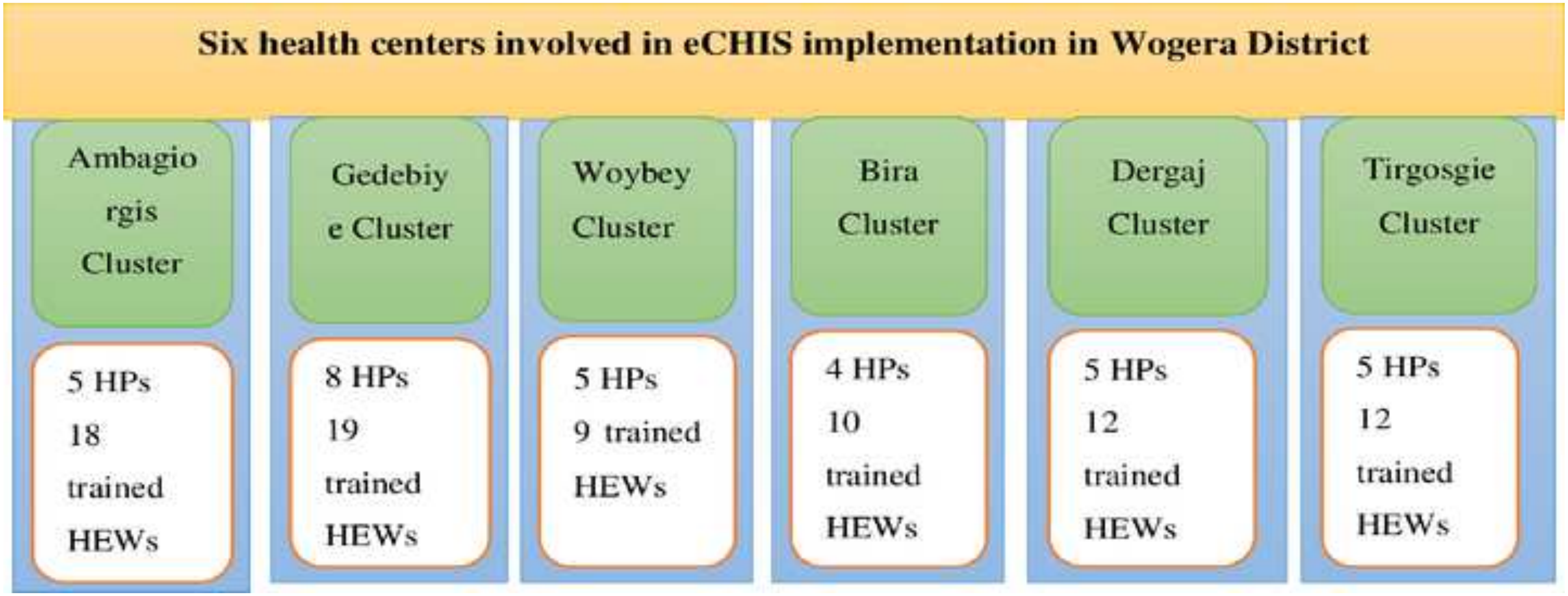

Figure Click here to access/download;Figure;Figure 5.tif

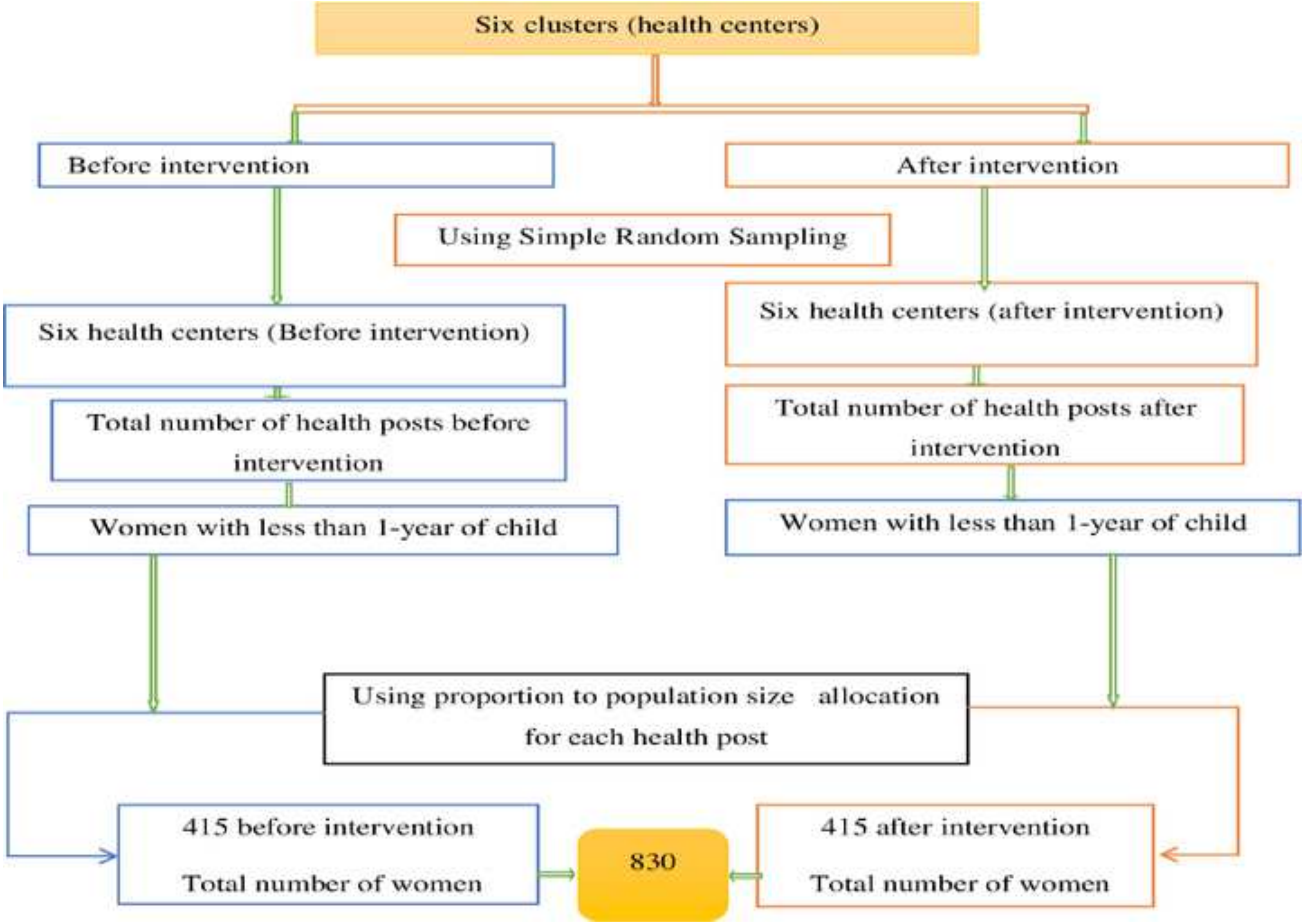

Click here to access/download;Figure;Figure 6.tif

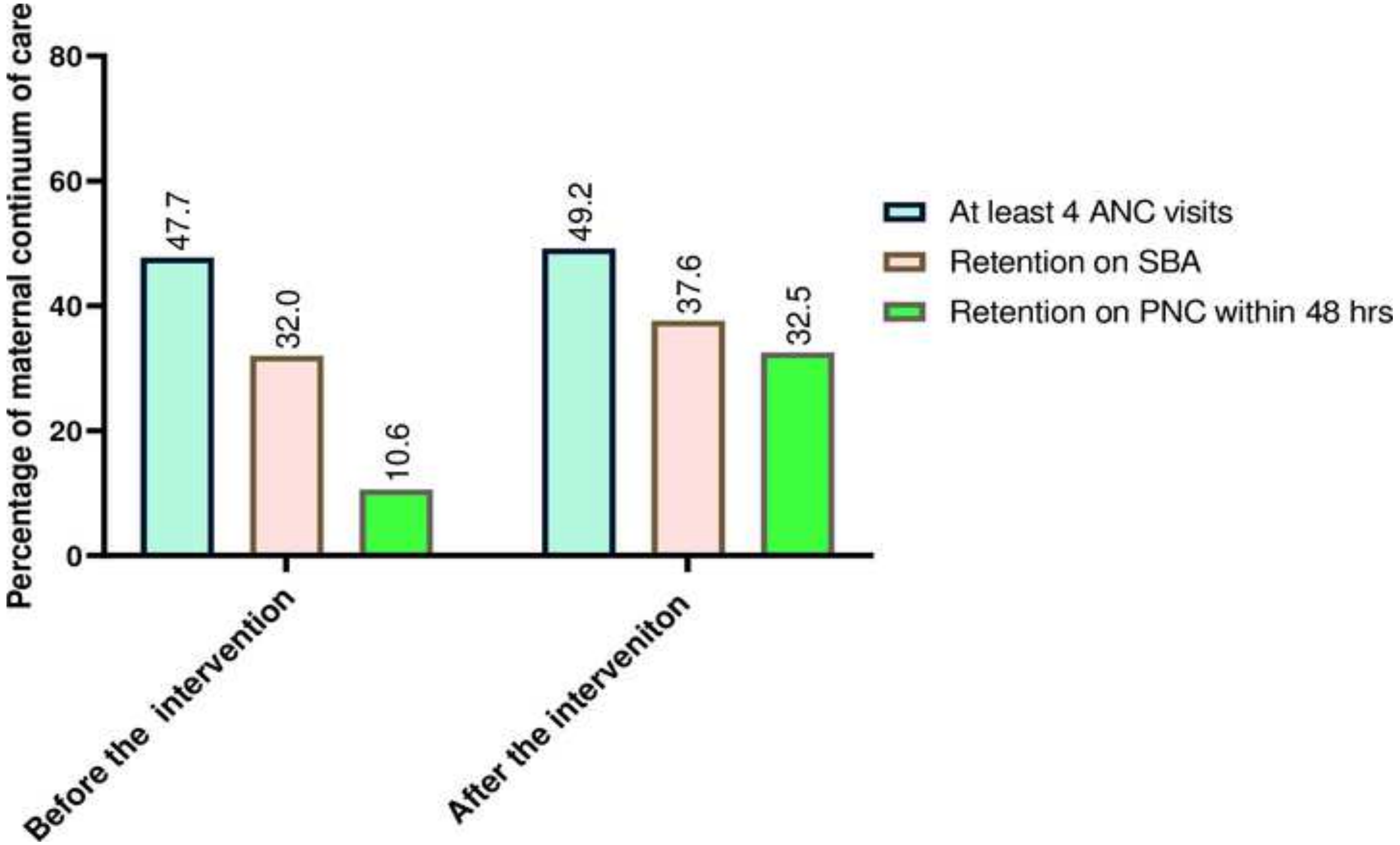

Point-by-point response to previous submission

Editor comments

	Comments	Authors response
1	Aspects of the study are not well described, including the study design and analysis methods	Thank you so much dear editor for the comments. The study design utilized in this study was mentioned on page 5, line 138-141 and the analysis method carried out was also found in page 8, line 238-244.
**2**	Data are presented in tables in ways that are difficult to follow	Thank you so much dear editor for the comments. This study was a pre-post study design and included participants at both points before and after the intervention. Data in the table were to show the participants characteristics particularly on the study variables and the numbers in the table were also explained in a textual form. Have a look on page 9 (line257-264); page 10 (line 270-273); page 11 (line 279-281); page 12 (line 285-286), and page 12 (line 292-306). Thank you so much!
**3**	The conclusions reached in the discussion, which focuses significantly on the role of health extension workers (HEW) do not seem to be supported by your results, which reveal that only a minority of participants had any exposure to HEWs.	Thank you dear editor for the comments. The study found that implementation of digital tool at community level using health extension workers (HEWs) was effective and the conclusion was based on this main findings and other covariates of participants found significant during analysis. In Ethiopia, HEWs are the last mile health workers at community level and the digital tool (eCHIS) was also designed and implemented considering their routine community level work architecture. Have a look on page 16, line 383-390. Thank you so much!
**4**	Lastly, if this paper is revised and submitted elsewhere, I strongly recommend a thorough copy edit and perhaps engaging a copy-editor	Thank you dear editor. Since the previous submission of the manuscript, we have been working on it and believe that the manuscript is improved substantially. Hope, we will learn more during revision of the current manuscript and improve it along the paths of publication. Thank you so much!
