# Supplementary material for: Digitalizing the community health information system improved women’s retention on the maternal continuum of care pathway in northwest Ethiopia: A quasi-experimental study
Source: PLoS One. 2025 Mar 10;20(3):e0312965. doi: 10.1371/journal.pone.0312965 (PMC11892869; doi:10.1371/journal.pone.0312965)
Supplement: Attachment — Submitted filename: Impact of digitalizing CHIS- Women health.docx [file pone.0312965.s003.docx]

Impact of digitalizing the community health information system on women’s retention in the maternal continuum of care pathway in northwest Ethiopia

Dear author, thank you for your effort to assess the impact of digitalizing the community health information system on women’s retention in the maternal continuum of care pathway in northwest Ethiopia. The manuscript has addressed an important topic, but incorporating the following comment can make the manuscript more understandable and repeatable.

Please give a line number; it makes it easy to comment and also helps determine whether the comments were addressed.

Some of the paragraphs lack references, and even all paragraphs are cited from a single paragraph. This shows adequate and relevant literature was not consulted. The manuscript can benefit from adequate analysis and synthesis of the literature in the field.

Please introduce each abbreviation in the first appearance in the manuscript. E.g. MMR, on the first line of page 3.

In the method section, please elaborate on the description of the study area. Include the regional state, zone, and other key socio demographic and economic features of the area. Also, the health service coverage, other infrastructure, including telecommunications .

It's more convenient to write the study design and population separately. It is wise to be specific while writing the study design, usually with a simple sentence. However, a description of the population may require a paragraph.

In the description of the intervention, the implementers and the implementation process were overemphasized (the government, the Amhara RHB).

Please describe the formula and the software used for sample size calculation. It's more appropriate if the sample size calculation and sampling technique come after the population section.

Please make sure the definition given to the outcome variable is clear and measurable. Also, in the references cited, the outcome was measured in the same way.

Do all the variables listed in the exposure variable were exposure? For the outcome. Do they have levels?

Clearly describe the types of data collection methods, did you prepare them or were they already used in similar studies previously? Clarify if the instrument is standard.

Also, write the last sentence of the data collection instrument section, as this sentence talks about data quality control and not about data collection tools.

On the ethical consideration part; why did you prefer to obtain verbal consent?

On the result part, please add the response rate.

What was your reference to categorize the age of the participant into three categories?

Page 10 of the maternal continuum of care before intervention was not clearly mentioned.

In table 4, log the binomial logistic regression model, add the p value.

On the discussion part, the paragraphs were too long. It is advisable to finalize the  paragraph in  5–6 lines addressing the same idea.

Give a heading for the limitations of the study.
