# Supplementary material for: Digitalizing the community health information system improved women’s retention on the maternal continuum of care pathway in northwest Ethiopia: A quasi-experimental study
Source: PLoS One. 2025 Mar 10;20(3):e0312965. doi: 10.1371/journal.pone.0312965 (PMC11892869; doi:10.1371/journal.pone.0312965)
Supplement: Attachment — Submitted filename: Review report.docx [file pone.0312965.s004.docx]

Review of manuscript titled "**Impact of digitalizing the community health information system on women’s retention in the maternal continuum of care pathway in northwest Ethiopia**" is a well-structured research article that addresses an important issue in public health.

**Here is a detailed review of the manuscript**:

**General Question or concerns**

1. **Retention Focus**: The focus is on "women’s retention," which is crucial but narrows the scope. Other related outcomes, such as quality of care, maternal or infant health outcomes, and overall system efficiency, are not explicitly mentioned.
2. **Digitalization Ambiguity**: The term "digitalizing" is broad. It could involve various processes, from simple data entry to complex system integrations. The title doesn’t specify what aspects of the community health information system are being digitalized, which could lead to varied interpretations. **Need operational definition**
3. The title implies a causal relationship between digitalization and retention in the maternal continuum of care, which might not be adequately supported depending on the study design (how digitalization can affect retention?? It could affect the quality of service??).

Retention could be due quality of service, midwife approach (CRC), free of cost and other.

4. On page 10, It says, “This study was conducted at Wogera district northwest Amhara, Ethiopia (Figure 1). The district has a total number 75 health centers and 404 health posts. During the study period, the number of surviving infants were 8675 [32].”

(Is it real number of health Facility in single Woreda/district?)

5. Ethical considerations: Verbal consent for pre-post why not written?
6. The Author mention as they didn’t receive any form of funding at the beginning and in the document, he/she mentioned the source of funding for this research work

Financial Disclosure: Author response: The author(s) received no specific funding for this work (Page 1), then page 23 said “**Funding:** This work would not be possible without the financial support from the Doris Duke Charitable Foundation (grant 2017187) through the University of Gondar.” Not contradicting??

**Specifically,**

**Title and Abstract:**

- **Title:** The title is clear and effectively summarizes the focus of the study.
- **Abstract:** The abstract provides a concise summary of the study's background, objectives, methods, results, and conclusions. However, the language could be improved for clarity, and minor grammatical corrections are needed. For instance, "Digitalizing primary healthcare using community health workers is one of the flagship agenda" should be revised to "Digitalizing primary healthcare using community health workers is a flagship agenda."

**Introduction:**

Consider rephrasing some sentences for clarity. For example, "Digitalizing primary healthcare using community health workers is one of the flagship agenda of the government of Ethiopia to improve maternal healthcare utilization" could be rephrased for better readability.

**Significance**

- **Relevance**: The title didn’t address a significant issue in maternal healthcare, particularly in low-resource settings like Ethiopia. Ensuring women’s retention in the maternal continuum of care and how Digitalizing is crucial for retention.

**Methods:**

- The study design, setting, and population are well-described. The use of a pre-post experimental design is appropriate for the study objectives.
- The statistical analysis section is clear, and the use of log-binomial logistic regression is appropriate for estimating the effect of the intervention.
- However, the manuscript could benefit from a more detailed description of the intervention itself. How was the electronic community health information system implemented? What were the specific components of the system?

**Population**: The design should ensure that the population studied is representative of the women who utilize the maternal care pathway in Northwest Ethiopia. This includes considering factors like rural vs. urban settings, socio-economic status, and literacy levels.

**Data Collection**

- **Data Sources**: The title suggests a reliance on data from a digitalized community health information system. It's important to clarify whether the data is reliable, complete, and accurately reflects the care continuum.
- **Methodology**: The methods of data collection should be detailed. This includes how data is extracted from the digital system, how retention is measured, and how the digitalization process itself is assessed.

**Results:**

- The results are presented clearly, with appropriate use of tables and statistical measures.
- The increase in maternal continuum of care from 10.6% to 32.53% is significant and well-supported by the data. Consider providing more context for the association between education level, pregnancy intention, and autonomy of women with the maternal continuum of care. This could help in understanding the broader implications of these findings.

**Discussion:**

- The discussion appropriately interprets the findings in the context of existing literature. The implications for policy and practice are well-articulated.
- It might be beneficial to discuss potential limitations of the study in more detail. For instance, were there any challenges in the implementation of the electronic community health information system that could have influenced the results?

**Conclusion:**

- The conclusion succinctly summarizes the key findings and their implications.
- **References:**
- The references appear to be relevant and up-to-date. Ensure that all references are correctly formatted according to the journal's guidelines and avoid self-citation ASAP.

**Language and Grammar:**

- The manuscript is generally well-written, but there are minor grammatical errors and awkward phrasing that should be corrected. For example, "Women’s retention on the paths of maternal continuum of care is still a significant challenge" could be rephrased as "Women's retention in the maternal continuum of care remains a significant challenge."

**Overall Recommendation:**

- **Medium revisions:** The manuscript is strong and contributes valuable insights into maternal health care in Ethiopia. Medium revisions to improve language clarity and provide additional details in the methods and discussion sections will enhance the manuscript's overall quality.

**Thank you.**

**NB. Don’t share my detail with Author!**

**Edosa Gutema (MPH/RH, PhD student)**

**Ethiopian Public Health Institute**

[**idoamante07@gmail.com**](mailto:idoamante07@gmail.com)

**+251911995580**
