# Supplementary material for: Digitalizing the community health information system improved women’s retention on the maternal continuum of care pathway in northwest Ethiopia: A quasi-experimental study
Source: PLoS One. 2025 Mar 10;20(3):e0312965. doi: 10.1371/journal.pone.0312965 (PMC11892869; doi:10.1371/journal.pone.0312965)
Supplement: Attachment — Submitted filename: Reviewers Response.docx [file pone.0312965.s006.docx]

**Date 18/09/2024**

**Rebuttal Letter**

**Subject: Question-by-question responses**

**Manuscript title:** Digitalizing the community health information system improved women’s retention on the maternal continuum of care pathway in northwest Ethiopia: a quasi-experimental study

**Manuscript number**: PONE-D-24-09283

**Authors:** Tesfahun Hailemariam, Asmamaw Atnafu, Lemma Derseh Gezie, and Binyam Tilahun

**Dear Editor,**

Thank you for taking the time to review and comment upon our manuscript, (Manuscript number: PONE-D-24-09283, Digitalizing the community health information system improved women’s retention on the maternal continuum of care pathway in northwest Ethiopia: a quasi-experimental study. We found the advice constructive and have incorporated the suggestions into our revised or cleaned manuscript. We have responded to each of the reviewers’ comments in our comment-by-comment responses below. We authors had been working on the manuscript since the submission of the paper and there is improvement from the first submission. It is our belief that the manuscript is substantially improved after all those comments and suggestions were addressed. We noted that the corrected texts at cleaned version may not exactly found on the page and line as mentioned by reviewers at the original commented manuscript because of the extensive revisions of each page and line. We want to inform you that the corrections made in the main document are indicated with track changes. Figure 1 and 2 in previous submission were deleted from the current submission as per the suggestion from journal requirement mentioned, as we cannot able to present written permission from the copyright holder to publish these figures.

Thank you again for your thoughtful comments. If anything we need to consider before publication, please let us know and learn! Thank you again!

On behalf of the group of authors

Sincerely,

Tesfahun Hailemariam

Point-by-point response

**Editor comments:**

|  | **Comments** | **Authors response** |
| --- | --- | --- |
| 1 | I critically reviewed your article entitled “Impact of digitalizing the community health information system on women’s retention in the maternal continuum of care pathway in northwest Ethiopia” which has the potential to add to the existing body of scientific knowledge, particularly in developing countries. However, there are some limitations in your article that need addressing before publication. | Thank you so much dear editor, we have addressed all your comments including reviewers’ comments. |
| 2 | There are several grammatical and typological errors that authors need to carefully review. | Thank you dear editor for very helpful comments. We carefully addressed your comments related to grammatical and typographic errors. |
| 3 | Authors should extensively format manuscripts based on PLOS ONE journal style, including file naming. Avoid unnecessary italicizing and capitalization throughout the manuscript. | Thank you dear editor. We have carefully considered your comments. |
| 4 | Make sure that your reference contains all the necessary details and PLOS ONE style. | Thank you dear editor. We have checked all the references according to the comments and PLOS ONE style. Thank you for your constructive comments. |

___________________________________________________________________________

**Reviewer #1**

|  | **Comments** | **Authors response** |
| --- | --- | --- |
| 1 | Dear author, thank you for your effort to assess the impact of digitalizing the community health information system on women’s retention in the maternal continuum of care pathway in northwest Ethiopia. The manuscript has addressed an important topic, but incorporating the following comment can make the manuscript more understandable and repeatable. | Thank you so much for your comments. We addressed all the comments given during the review process. Have a look on the entire document, please. |
| 2 | Please give a line number; it makes it easy to comment and also helps determine whether the comments were addressed. | Thank you very much for the constructive comments. We provided a line number in the entire document. |
| 3 | Some of the paragraphs lack references, and even all paragraphs are cited from a single paragraph. This shows adequate and relevant literature was not consulted. The manuscript can benefit from adequate analysis and synthesis of the literature in the field. | We reviewed the manuscript and included essential citations for the current work. Would you please, have a look on the introduction section, Page 3, line 63-67, page 4, line 94-96, page 4, line 111-114? |
| 4 | Please introduce each abbreviation in the first appearance in the manuscript. E.g. MMR, on the first line of page 3. | Thank you so much dear reviewer for your valuable comments. We revised the entire document and introduced all abbreviations in their first appearance. Thank you so much! |
| 5 | In the method section, please elaborate on the description of the study area. Include the regional state, zone, and other key socio demographic and economic features of the area. Also, the health service coverage, other infrastructure, including telecommunications. | Thank you so much dear reviewer for the very valuable comments. We enriched the method section according to your comments. Please have a look on the method section, page 5, line 139-149 |
| 6 | It's more convenient to write the study design and population separately. It is wise to be specific while writing the study design, usually with a simple sentence. However, a description of the population may require a paragraph. | Thank you very much dear reviewer. We separated study design and population. Have a look at on page 6, 157-162 |
| 7 | In the description of the intervention, the implementers and the implementation process were overemphasized (the government, the Amhara RHB). | Thank you dear reviewer for you suggestion. However, this intervention was led by RHB in collaboration with University of Gondar in Ethiopia to facilitate and the process of digital tool(eCHIS) implementation in Wogera district which was a demonstration site for eCHIS implementation in the region. We, authors, honestly narrated all the documented facts in the intervention description section to indicate that how the implementation was going on during the entire period. Thank you so much. |
| 8 | Please describe the formula and the software used for sample size calculation. It's more appropriate if the sample size calculation and sampling technique come after the population section. | Thank you so much. We described the software we used during sample size determination. We also move the sample size and sampling technique section according to the recommendation. Please have a look on page 6. |
| 9 | Please make sure the definition given to the outcome variable is clear and measurable. Also, in the references cited, the outcome was measured in the same way. | Thank you dear reviewer for the valuable comments. The outcome in the current study was from ANC1 to PNC48hrs on the maternal healthcare continuum. We categorized women who fulfil all the required indicators such as ANC1, ANC4+ SBA+PNC48h, otherwise, no. The reference we cited also supports categorization of outcome variable we measured. Thank you so much! |
| 10 | Do all the variables listed in the exposure variable were exposure? For the outcome. Do they have levels? | Thank you dear reviewer for the comments. In the exposure section, we included variables from socio-demographic and reproductive characteristics of participants and we levelled them by consulting literature in the area. |
| 11 | Clearly describe the types of data collection methods, did you prepare them or were they already used in similar studies previously? Clarify if the instrument is standard. | Thank you so much dear reviewer. We addressed your comments in the revised version of the manuscript. Have a look on the data collection section, please. |
| 12 | Also, write the last sentence of the data collection instrument section, as this sentence talks about data quality control and not about data collection tools. | Thank you dear reviewer for the valuable comments. We corrected it according to your comments. Have a look on the data collection instrument section, please. |
| 13 | On the ethical consideration part; why did you prefer to obtain verbal consent? | Thank you dear reviewer for the valuable comments. In the current study, a limited literacy condition was a reason to authors to prefer verbal consent, more than 55% of participants did not attend education in the current study. We employed the data collection after a detailed explanation and understanding on the research objective and its importance to public health. Moreover, the participants had an opportunity to understand the study objective and its relevance. |
| 14 | On the result part, please add the response rate. | Thank you dear reviewer for the relevant comments to improve our work. The response rate was appeared in the first sentence of the result section. Have a look on the page 10, line 308, please. |
| 15 | What was your reference to categorize the age of the participant into three categories? | Thank you dear reviewer for the question, We, authors, categorized age as it appeared in the manuscript based on the previous literature. Demographic and health survey program often apply these age groupings to examine key indicators such as reproductive health, adolescent health, maternal and child health conditions. Moreover, Ethiopian demographic and health survey often uses this age categories to study maternal and child healthcare utilization. Thank you so much! |
| 16 | Page 10 of the maternal continuum of care before intervention was not clearly mentioned. | Thank you dear reviewer for the critical insight. We corrected the maternal continuum of care in the result section where it should appear. Have a look on the maternal continuum of care section, please. Thank you so much! |
| 17 | In table 4, log the binomial logistic regression model, add the p value. | Thank you so much dear reviewer for the very constructive comments. We addressed your comments and included the p-value for all the variables in the table 4. Have a look on the revised version of manuscript at table 4, please. Thank you again! |
| 18 | On the discussion part, the paragraphs were too long. It is advisable to finalize the paragraph in 5–6 lines addressing the same idea. | Thank you dear reviewer for your constructive comments. The extension of sentences in a given paragraph was that due to various justifications for a variable associated in the study. However, we had a revised it according to your comments. Thank you so much! |
| 19 | Give a heading for the limitations of the study. | Thank you so much dear for your valuable insight. We have included limitations, strengths and conclusion sections in the manuscript in a separate headings. We Thank you a lot for your educational comments. Thank you again! |

**Reviewer #2**

|  | **Comments** | **Authors response** |
| --- | --- | --- |
| 1 | The study effectively demonstrates the positive impact of the electronic Community Health Information System (eCHIS) on maternal health service utilization. However, there is a notable disconnect between the study's introduction, which emphasizes the need for research on the role of trained Community Health Workers (CHWs) in utilizing eCHIS, and the results and conclusions, which do not sufficiently address this focus. To improve coherence, I recommend that the authors either integrate findings related to the CHWs' contributions into the results and conclusions or adjust the introduction to better align with the broader scope of the analysis presented. | Thank you dear reviewer for your very constructive comments. We have considered your comments and revised our manuscript accordingly. Have a look on the revised version of the manuscript page 4-5, line 123-129 |
| 2 | Furthermore, it is important to acknowledge the absence of a control group, as this limitation raises concerns that the observed changes might not solely result from the eCHIS implementation, affecting the study's generalizability. | Thank you so much dear reviewer for your constructive comments. We have revised out manuscript according to your comments. Have a look on the limitation section in the revised manuscript, please, page 18, line 439-440 |
| 3 | To enhance transparency and reproducibility please specify the exact sampling interval used in the systematic random sampling process. | Thank you so much dear reviewer for your valuable comments. We have addressed your comments and have a look on the sampling procedure section, please, page 6-7, line 186-192 |
| 4 | Lastly, I suggest updating outdated references to include more recent research, which would reinforce the study's relevance and academic rigor. | Thank you so much dear reviewer for your comments. He have enriched the revised manuscript addressing all the comments and included relevant references to the current study. |

**Reviewer #3**

|  | **Comments** | **Authors response** |
| --- | --- | --- |
| 1 | Retention Focus: The focus is on "women’s retention," which is crucial but narrows the scope. Other related outcomes, such as quality of care, maternal or infant health outcomes, and overall system efficiency, are not explicitly mentioned. | Thank you dear reviewer for your direction to address other outcomes. However, this particular study focused to evaluate the impact of eCHIS on maternal continuum of care in the intervention district and other adjacent comparator district. Yes, it is possible to consider them the intervention effect on the quality of healthcare and child health outcomes. Hopefully, we will consider them in our next consecutive works. |
| 2 | Digitalization Ambiguity: The term "digitalizing" is broad. It could involve various processes, from simple data entry to complex system integrations. The title doesn’t specify what aspects of the community health information system are being digitalized, which could lead to varied interpretations. Needs proper definition. | Thank you dear reviewer for your constructive comments. We addressed your comments. Have a look on the operational deffiniont section, page 10, line 294-297.  eCHIS is initially deployed to digitize the PHC units where manual or CHIS is being applied. In digitalization process CHIS is replaced by eCHIS so that HEWs use tablet withHEWS application, focal person uses Apps with focal person application and MWs use tablet with MWs application to link community activities with catchment health facilities. Thus, this study was aimed to determine the effect of eCHIS implementation in Wogera district, a demonstration district selected by MoH and ARHB by providing technical support from University of Gondar. Thus, we applied before after intervention study, a quasi-experimental study design to evaluate its effectiveness on maternal continuum of care. Thank you so much! |
| 3 | The title implies a causal relationship between digitalization and retention in the maternal continuum of care, which might not be adequately supported depending on the study design (how digitalization can affect retention?? It could affect the quality of service??).  Retention could be due quality of service, midwife approach (CRC), free of cost and other. | The assumption in eCHIS was to tackle the challenges that existed with manual health data generation, use, and service provision using CHIS in primary health units in Ethiopia. eCHIS is one of the technology platforms that uses mobile applications for HEWs to enhance routine health data management and use and service provision. In the entire period of the study, the distict was implementing eCHIS ad a digital platform for data capturing, use and service provision. We hypothesized that e-CHIS implementation improves improve the quality of health data production, data use and service provision by transforming the culture of health post-data management practice through digitalizing. Thus, we utilized a quasi-experimental study design to see the effect of digitalization (eCHIS) on maternal continuum of care using pre post intervention data by controlling other confounders. Thank you for your question and we learned a lot! |
| 4 | On page 10, It says, “This study was conducted at Wogera district northwest Amhara, Ethiopia (Figure 1). The district has a total number 75 health centers and 404 health posts. During the study period, the number of surviving infants were 8675 [32].”  (Is it real number of health Facility in single Woreda/district?) | Thank you so much dear reviewer for your critical insight. We corrected since we missed some information of the zone during the write up of the manuscript. Have a look on the study setting secton of the study, please, page 5, line 145-159.  Thank you again. |
| 5 | Ethical considerations: Verbal consent for pre-post why not written? | Thank you dear reviewer for the valuable comments. In the current study, a limited literacy condition was a reason to authors to prefer verbal consent, more than 55% of participants did not attend education in the current study. We employed the data collection after a detailed explanation and understanding on the research objective and its importance to public health. Moreover, the participants had an opportunity to understand the study objective and its relevance. |
| 6 | The Author mention as they didn’t receive any form of funding at the beginning and in the document, he/she mentioned the source of funding for this research work  Financial Disclosure: Author response: The author(s) received no specific funding for this work (Page 1), then page 23 said “Funding: This work would not be possible without the financial support from the Doris Duke Charitable Foundation (grant 2017187) through the University of Gondar.” Not contradicting?? | Thank you dear reviewer for your valuable comments. Sorry for the contradicting contents in the financial statement. We corrected it in the manuscript and system during submission. Thank you so much! |
| 7 | Title: The title is clear and effectively summarizes the focus of the study. | Thank you so much! |
| 8 | Abstract: The abstract provides a concise summary of the study's background, objectives, methods, results, and conclusions. However, the language could be improved for clarity, and minor grammatical corrections are needed. For instance, "Digitalizing primary healthcare using community health workers is one of the flagship agenda" should be revised to "Digitalizing primary healthcare using community health workers is a flagship agenda." Consider rephrasing some sentences for clarity. For example, "Digitalizing primary healthcare using community health workers is one of the flagship agenda of the government of Ethiopia to improve maternal healthcare utilization" could be rephrased for better readability. | Thank you so much dear reviewer for your constructive comments. We have been working on the manuscript since its submission and we had incorporated all reviewers’ comments to enhance its quality for publication. Thank you so much! |
| 9 | Relevance: The title didn’t address a significant issue in maternal healthcare, particularly in low-resource settings like Ethiopia. Ensuring women’s retention in the maternal continuum of care and how Digitalizing is crucial for retention. | Thank you dear reviewer for your insightful comments. We revised our title according to your comments. Have a look on the title of the revised manuscript, please. Thank you so much! |
| 10 | The study design, setting, and population are well-described. The use of a pre-post experimental design is appropriate for the study objectives. | Thank you so much! |
| 11 | The statistical analysis section is clear, and the use of log-binomial logistic regression is appropriate for estimating the effect of the intervention. However, the manuscript could benefit from a more detailed description of the intervention itself. How was the electronic community health information system implemented? What were the specific components of the system? | Thank you so much dear reviewer. We have provided detailed description on the intervention section that narrates the components delivered during the intervention. Have a look on the intervention description section on Method section, please, page 7-8, line 205-232. Thank you so much! |
| 12 | Population: The design should ensure that the population studied is representative of the women who utilize the maternal care pathway in Northwest Ethiopia. This includes considering factors like rural vs. urban settings, socio-economic status, and literacy levels. | Thank you so much dear reviewer for your constructive comments. This study considers child bearing women who had less than 1 year of child in the two districts in northwest Ethiopia. As the study consumes simple random techniques in getting participants, those urban and rural women had equal chance of involvement in the study as both districts have rural and urban kebeles, the smallest administrative unit in Ethiopia. Thank you so much! |
| 13 | Data Sources: The title suggests a reliance on data from a digitalized community health information system. It's important to clarify whether the data is reliable, complete, and accurately reflects the care continuum. | Thank you dear reviewer. This study relies on community based study using women in both intervention and comparator districts following all necessary scientific methods. Thank you so much! |
| 14 | Methodology: The methods of data collection should be detailed. This includes how data is extracted from the digital system, how retention is measured, and how the digitalization process itself is assessed. | Thank you so much dear reviewer. This study depends on pre-post study design and participants at both study points were from the intervention and comparator districts. We utilized the survey data at household level via face to face interview of women for impact assessment of eCHIS on maternal continuum of care. Thank you so much! |
| 15 | The results are presented clearly, with appropriate use of tables and statistical measures. | Thank you so much! |
| 16 | The increase in maternal continuum of care from 10.6% to 32.53% is significant and well-supported by the data. Consider providing more context for the association between education level, pregnancy intention, and autonomy of women with the maternal continuum of care. This could help in understanding the broader implications of these findings. | Thank you so much dear reviewer. We revised and provided more context on the intervention and associated factors in the study in the discussion section. Thank you so much! |
| 17 | The discussion appropriately interprets the findings in the context of existing literature. The implications for policy and practice are well-articulated. | Thank you so much dear reviewer! |
| 18 | It might be beneficial to discuss potential limitations of the study in more detail. For instance, were there any challenges in the implementation of the electronic community health information system that could have influenced the results? | Thank you so much dear reviewer. We had revised the limitation section according to your comments.  Have a look on the limitation section in the revised manuscript, please. |
| 19 | The conclusion succinctly summarizes the key findings and their implications. | Thank you so much! |
| 20 | The references appear to be relevant and up-to-date. Ensure that all references are correctly formatted according to the journal's guidelines and avoid self-citation ASAP. | Thank you so much. We have checked all the references for their up-to-date and in accordance to journal guideline. |
| 21 | The manuscript is generally well-written, but there are minor grammatical errors and awkward phrasing that should be corrected. For example, "Women’s retention on the paths of maternal continuum of care is still a significant challenge" could be rephrased as "Women's retention in the maternal continuum of care remains a significant challenge." | Thank you so much dear reviewer for your very valuable comments. We had worked on your comments and we believe the manuscript has substantially improved. Thank you so much! |
| 22 | Medium revisions: The manuscript is strong and contributes valuable insights into maternal health care in Ethiopia. Medium revisions to improve language clarity and provide additional details in the methods and discussion sections will enhance the manuscript's overall quality. | Thank you so much dear reviewer for your educational comments. We addressed your comments and provided additional details on background, methods, and discussion section. Have a look on the revised manuscript, please.  Thank you so much! |

**Reviewer #4**

|  | **Comments** | **Authors response** |
| --- | --- | --- |
| 1 | The abstract should not exceed 300 words | Thank you dear reviewer for your comments. We followed Plos One guideline and the word count do not exceed the acceptable number of counts. Thank you so much! |
| 2 | The study design and study population are not well described. | Thank you so much dear reviewer for your comments. We revised the manuscript according to your comments and now the manuscript has been substantially improved. |
| 3 | Did you differentiate when to use simple random sampling and systematic random sampling? | Thank you so much dear reviewer. We applied simple random sampling to get health centers at both districts whereas systematic simple random sampling to get women at household level from defined number of listed women at each health posts level where HEWs archive the number of women at kebele who seek maternal and child health services. Thank you so much! |
| 4 | What is wrong if we use simple random sampling to select study participants? | Thank you dear reviewer for your interesting question. Possible, however, in our study we applied systematic simple random sampling preferring that there were a defined frame of women at health post where HEWs provide sufficient information about mothers and their lists too. Thank you so much! |
