# Supplementary material for: Digitalizing the community health information system improved women’s retention on the maternal continuum of care pathway in northwest Ethiopia: A quasi-experimental study
Source: PLoS One. 2025 Mar 10;20(3):e0312965. doi: 10.1371/journal.pone.0312965 (PMC11892869; doi:10.1371/journal.pone.0312965)
Supplement: Attachment — Submitted filename: Reviewer.docx [file pone.0312965.s007.docx]

**Subject: Review of Revised Manuscript PONE-D-24-09283R1**

Dear Editor,

Thank you for the opportunity to review the revised version of Manuscript Number PONE-D-24-09283R1, titled "Digitalizing the community health information system improved women’s retention on the maternal continuum of care pathway in northwest Ethiopia: a quasi-experimental study."

I appreciate the authors' efforts in addressing the comments and suggestions provided during the initial review. Upon evaluating the revised manuscript, I have noted that the authors have effectively incorporated the feedback and made significant improvements.

Thank you again for involving me in this process, and I look forward to seeing the paper published.

Eyob Getachew

University of Gondar institute of IPH department of Health Promotion and Health Behavior
