# Supplementary material for: Digitalizing the community health information system improved women’s retention on the maternal continuum of care pathway in northwest Ethiopia: A quasi-experimental study
Source: PLoS One. 2025 Mar 10;20(3):e0312965. doi: 10.1371/journal.pone.0312965 (PMC11892869; doi:10.1371/journal.pone.0312965)
Supplement: Attachment — Submitted filename: Response to Reviewers.docx [file pone.0312965.s008.docx]

**Date 03/02/2025**

**Rebuttal Letter**

**Subject: Point-by-point responses**

**Manuscript title:** Digitalizing the community health information system improved women’s retention on the maternal continuum of care pathway in northwest Ethiopia: a quasi-experimental study

**Manuscript number**: PONE-D-24-09283R1

**Authors:** Tesfahun Hailemariam, Asmamaw Atnafu, Lemma Derseh Gezie, and Binyam Tilahun

**Dear Editor,**

Thank you for taking the time to review and comment upon our manuscript, (Manuscript number: PONE-D-24-09283R1, Digitalizing the community health information system improved women’s retention on the maternal continuum of care pathway in northwest Ethiopia: a quasi-experimental study. We found the comments were constructive and we addressed all the comments raised during revision. We have responded to each the editor’s and reviewer’s comments in our point by point- response below. We authors had been working on the manuscript since the submission of the paper and there is improvement from the first submission. In the previous version, table 1 and table 2 were repeated in both text and table format, we ask apology for that and now we made correction on the current cleaned version of the manuscript. It is our belief that the manuscript is substantially improved after all those comments and suggestions were addressed. Thank you again for your thoughtful comments. If anything we need to consider before publication, please let us know and learn! Thank you again!

On behalf of the group of authors

Sincerely,

Tesfahun Hailemariam

Point-by-point response

______________________________________________________________________________

**Editor comments**

**Authors:** Dear Editor, we thank you for comments and requesting us to submit a revised version. We hope that we have addressed all the mentioned comments raised by dear editor. We believe that the comments from editor were addressed; if there is any issue that is not addressed, please let us know and learn!

Thank you so much!

Point-by-point response to previous submission

___________________________________________________________________________

**Editor comments**

|  | **Comments** | **Authors response** |
| --- | --- | --- |
| 1 | There are several inconsistencies between the introduction and findings that authors need to carefully review. | Thank you dear editor for your very constructive comments. We have carefully addressed the study outcome (maternal continuum of care) and the contents appeared in the introduction section. |
| **2** | Make sure that your reference contains all the necessary details and PLOS ONE style. | Thank you again dear editor for the suggestion. We checked the reference sections to ensure with PLOS ONE style. |
| **3** | Authors should acknowledge the limitation and elaborate the sampling at this time to revise the manuscript. | Thank you dear editor for your very insightful comments. In the discussion section, we have acknowledged the limitation o of the study and revised out manuscript according to your comments. |

___________________________________________________________________________

**Reviewers’ comments #3**

|  | **Comments** | **Authors response** |
| --- | --- | --- |
| 1 | How digitalization affect retention of mother? It might have effect on quality of service (e.g. timing, retraction of data, for data loss) with can affect the retention. However, How digitalization can directly have impact on maternal retention? | Thank you so much dear reviewer for your insightful comment. In digitalization of community health information system, HEWs, focal persons, and a midwifery or nurse involved in the referral linkage and thus they provide maternal health services in integration where every pregnant woman gets a chance to be registered in the application and receive maternal health services. Moreover, digitalization enables HEWs to provide maternal health services at community level and help them to link pregnant women within the catchment health facilities for their respective maternal healthcare follow up, ANC visits, facility delivery and PNC visits and that enhances women retention on their respective maternity care continuum, as the system provides all MNCH program in the implementation dispirit. |
| **2** | This is a quasi experimental study, and the authors report 100% response rate? Could this be real in context of Ethiopia, specially in Amhara region with many conflicts and displaced populations? | Thank you dear reviewer again for your very interesting comment. We applied a quasi-experimental study design for this particular study. At the baseline and endline, we used the same population, i.e., all women in the study districts who gave birth within the past one year prior to data collection were the source population, and randomly selected women who were permanent residents of the selected kebeles and willing to participate in the study were the study population. Thus, at both study ends, women in the clusters were considered to be study participants and we draw the study participants through proportional allocation to the clusters in the district before and after the implementation of the program. Thus, according to the estimated sample size, we included a total of 830 women which considers the two samples before and after the study (n1=n2=415). Thank you so much again! |

**Thank you so much!**
